# Guanine Nucleotide Exchange Factors and Small GTPases: Their Regulation and Functions, Diseases, and Therapeutic Targets

**DOI:** 10.1002/mco2.70362

**Published:** 2025-09-27

**Authors:** Zexing Lin, Chujun Ni, Haiyang Jiang, Huan Yang, Liting Deng, Peizhao Liu, Xuanheng Li, Yilong Yu, Weijie Li, Runnan Wang, Bo Liao, Jiaqi Kang, Juanhan Liu, Xiuwen Wu, Jianan Ren, Yun Zhao

**Affiliations:** ^1^ Department of General Surgery Nanjing BenQ Medical Center The Affiliated BenQ Hospital of Nanjing Medical University Nanjing China; ^2^ Department of Surgical Research Laboratory BenQ Medical Center The Affiliated BenQ Hospital of Nanjing Medical University The Clinical Translational Research Center For Surgical Infection and Immunity of Nanjing Medical University Nanjing Jiangsu China; ^3^ School of Medicine Southeast University Nanjing China; ^4^ Research Institute of General Surgery Affiliated Jinling Hospital Medical School of Nanjing University Nanjing China; ^5^ Department of Critical Care Medicine The Affiliated Jiangning Hospital with Nanjing Medical University Nanjing Jiangsu China

**Keywords:** cancer metastasis, guanine nucleotide exchange factors, GEF, microtubule dynamics, neurodegenerative diseases, Rho signaling, small GTPases

## Abstract

Guanine nucleotide exchange factors (GEFs) and their small GTPase substrates constitute a fundamental regulatory system that governs diverse cellular processes, including cytoskeletal dynamics, membrane trafficking, and transcriptional regulation. Since their discovery, GEFs have been recognized as molecular switches that activate small GTPases by catalyzing GDP‐to‐GTP exchange, thereby playing pivotal roles in cellular signaling and homeostasis. Despite extensive research, key gaps remain in understanding the spatiotemporal regulation of GEF isoforms, their functional redundancy in disease, and the development of isoform‐specific drugs. This review examines the regulatory mechanisms and physiological roles of GEFs, highlighting their growing potential as therapeutic targets. We explore the phylogenetic classification of GEFs into major families (Ras, Rho, Rab, and ArfGEFs) and their regulatory networks, which encompass subcellular localization, posttranslational modifications, and scaffolding interactions. Special emphasis is placed on GEF–H1, a microtubule‐regulated RhoGEF, and its roles in cytoskeletal remodeling, cancer metastasis, and immune responses. We also examine GEF dysregulation in diseases like cancer, neurodegeneration, and cardiovascular disorders, and assess current therapies, such as small‐molecule inhibitors and emerging PROTAC technology. This review connects GEF biology with clinical applications by combining basic research with translational insights, providing guidance for precision medicine and novel therapeutic strategies targeting GEF‐related diseases.

## Graphical abstract

This review comprehensively examines the regulatory mechanisms and physiological roles of guanine nucleotide exchange factors (GEFs) and their small GTPase substrates, highlighting their significance in cellular processes and disease pathogenesis. We discuss the classification, core regulatory mechanisms, and dysregulation of GEFs in cancer, neurodegenerative diseases, and immune disorders. Additionally, we evaluate current therapeutic strategies targeting GEFs, including small‐molecule inhibitors and emerging technologies like PROTACs, providing insights for future drug development.

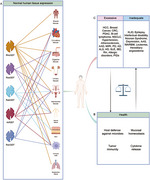

## 1 Introduction

Guanine nucleotide exchange factors (GEFs) and their small GTPase substrates constitute a fundamental regulatory system that governs diverse cellular processes, including cytoskeletal dynamics, membrane trafficking, and transcriptional regulation [1, 2] (Table 1). Since the discovery of the Rho gene family in 1985, the field has witnessed exponential growth, revealing the critical roles of GEFs as molecular switches that activate small GTPases by catalyzing GDP‐to‐GTP exchange [3]. Over the past decades, extensive research has elucidated the phylogenetic classification of GEFs into distinct families (Ras, Rho, Rab, and ArfGEFs) and their intricate regulatory mechanisms involving subcellular localization, posttranslational modifications, and scaffolding interactions [4, 5]. Current studies highlight their emerging significance in human diseases, positioning GEFs as promising therapeutic targets for cancer, neurodegenerative disorders, and immune dysregulation [6].

**TABLE 1:** Classification and functional characteristics of the GEFs family.

Classification	G protein	Functions	Member	Domain	Disease	References
RasGEFs (SOS family, RasGRP family, EPAC family)	Ras superfamily (H/K/N‐Ras, Rap)	Cell proliferation, differentiation, and survival (e.g., MAPK pathway)	SOS1/2, RasGRP, EPAC	REM (Ras exchange module), CDC25 domain	Cancer, leukemia, cardiovascular diseases	[7, 8, 9]
RhoGEFs (Dbl family, DOCK family	Rho family (RhoA, Rac1, Cdc42)	Cytoskeleton reorganization, migration, and polarity	Vav1/2/3, Tiam1/2, Dbl	DH + PH domain	Cancer metastasis, Immune deficiency, neurodegeneration	[2, 10, 11, 12, 13, 14]
RabGEFs (TRAPP complex, DENND family, Vps9 domain family	Rab family (Rab5, Rab7, etc.)	Vesicle transport, membrane trafficking	TRAPP, DENND1A, Rabex‐5	Vps9 domain	Abnormal bone development, ovarian cancer, neurodevelopmental disorder	[15, 16]
ArfGEFs (BIG/GBF subfamily, cytohesin subfamily, EFA subfamily	Arf family (Arf1, Arf6)	Golgi transport and endocytosis	BIG1/2, ARNO/cytohesin, EFA6	Sec7 domain	Infection, inflammation, cancer invasion	[17, 18, 19, 20]
RanGEFs	Ran	Nuclear transport and mitosis	RCC1	Chromatin binding domain	Chromosome instability	[21, 22, 23]

Abbreviations: BIG: Brefeldin A‐inhibited guanine nucleotide‐exchange; DENND: differentially expressed in normal and neoplastic cells domain; DOCK: dedicator of cytokinesis; EPAC: exchange protein directly activated by camp; RCC1: regulator of chromosome condensation 1; TRAPP: transport protein particle; VPS9: vacuolar protein sorting 9.

This review is motivated by the need to consolidate the rapidly expanding knowledge on GEF biology, with a particular focus on GEF–H1, a microtubule‐regulated Rho GEF [24]. Although significant advances have been made in understanding GEF–H1's roles in cytoskeletal remodeling and disease pathogenesis, critical knowledge gaps persist. These include the functional characterization of alternatively spliced isoforms and the integration of its microtubule‐dependent and ‐independent activation pathways. While GEF–H1 dysregulation has been implicated in cancer metastasis, immune responses, and vascular pathologies [25, 26], key aspects remain underexplored: the compartmentalized signaling of its isoforms, their context‐dependent functional redundancy in disease states, and the therapeutic implications of isoform‐specific regulation. Addressing these questions is essential for developing isoform‐specific pharmacological agents and advancing precision medicine.

To address these gaps, this review will first provide a comprehensive classification of GEF families and their coevolution with small GTPases, emphasizing structural and functional diversity. It will then delve into the core regulatory mechanisms of GEF–GTPase systems, including the structural basis of nucleotide exchange, spatiotemporal control via microtubules and membranes, and posttranslational modifications. Subsequent sections will explore the physiological functions of GEF networks in cell motility, membrane trafficking, and immune regulation, followed by an in‐depth analysis of their dysregulation in cancer, neurodegeneration, cardiovascular diseases, and immunological disorders. The review will conclude with an evaluation of current therapeutic strategies, such as small‐molecule inhibitors and emerging PROTAC technology, while outlining future research directions to overcome existing challenges.

By integrating fundamental research with translational applications, this review aims to bridge the gap between GEF biology and clinical potential. The logical progression from molecular mechanisms to physiological roles and therapeutic targeting provides a cohesive framework for understanding GEFs as central regulators of cellular signaling and disease. This synthesis not only highlights the complexity of GEF–GTPase systems but also underscores their untapped potential for innovative treatments in precision medicine.

## 2 Classification and Evolution of GEFs and Small GTPases

**FIGURE 1:**
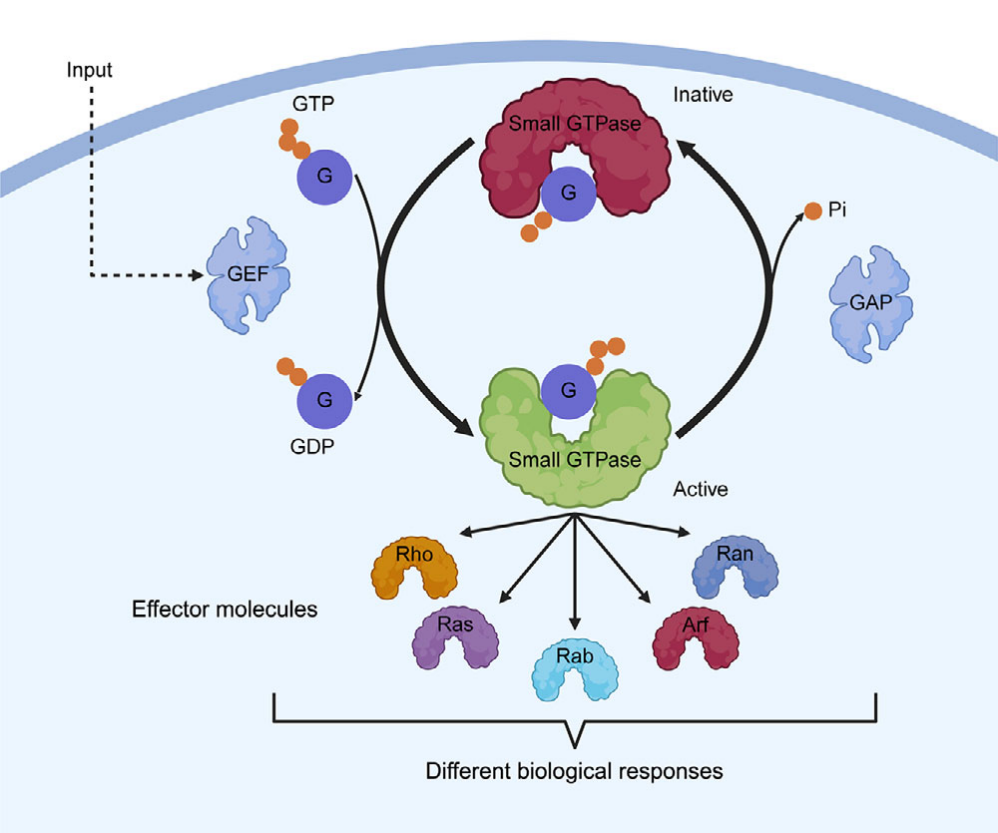
Small GTP protease activity cycle. GTPases function as molecular switches by cycling between an active GTP‐bound (“on”) state and an inactive GDP‐bound (“off”) state, a process regulated by GAPs (GTPase‐activating proteins) and GEFs. GAPs accelerate GTP hydrolysis, converting GTP to GDP and inorganic phosphate (Pi), thereby switching the GTPase off. In contrast, GEFs promote GDP release, enabling GTP rebinding and reactivation of the protein. These transitions involve conformational changes in the GTPase's switch regions, where GTP stabilizes an active conformation that engages downstream effectors, while GDP induces an inactive state. Figures created with BioRender.com.

GEFs are a class of crucial regulatory proteins that play central roles in diverse cellular physiological processes by specifically activating small GTPases (including Ras, Rho, Rab, Arf, and Ran superfamily members) [5]. These proteins catalyze the transition of small GTPases from GDP‐bound to GTP‐bound states through precise molecular mechanisms. First, they recognize and bind to GDP‐bound small GTPases, inducing conformational changes that promote GDP dissociation. Next, they facilitate GTP binding due to the higher intracellular GTP concentration. Finally, the activated GTPase–GTP complex is released to trigger downstream signaling cascades [1, 27] (Figure 1). GEFs exhibit highly specific expression patterns and substrate selectivity, and their dysfunction is closely associated with various major diseases, including cancer, neurodegenerative disorders, and immune system dysregulation.

**FIGURE 2:**
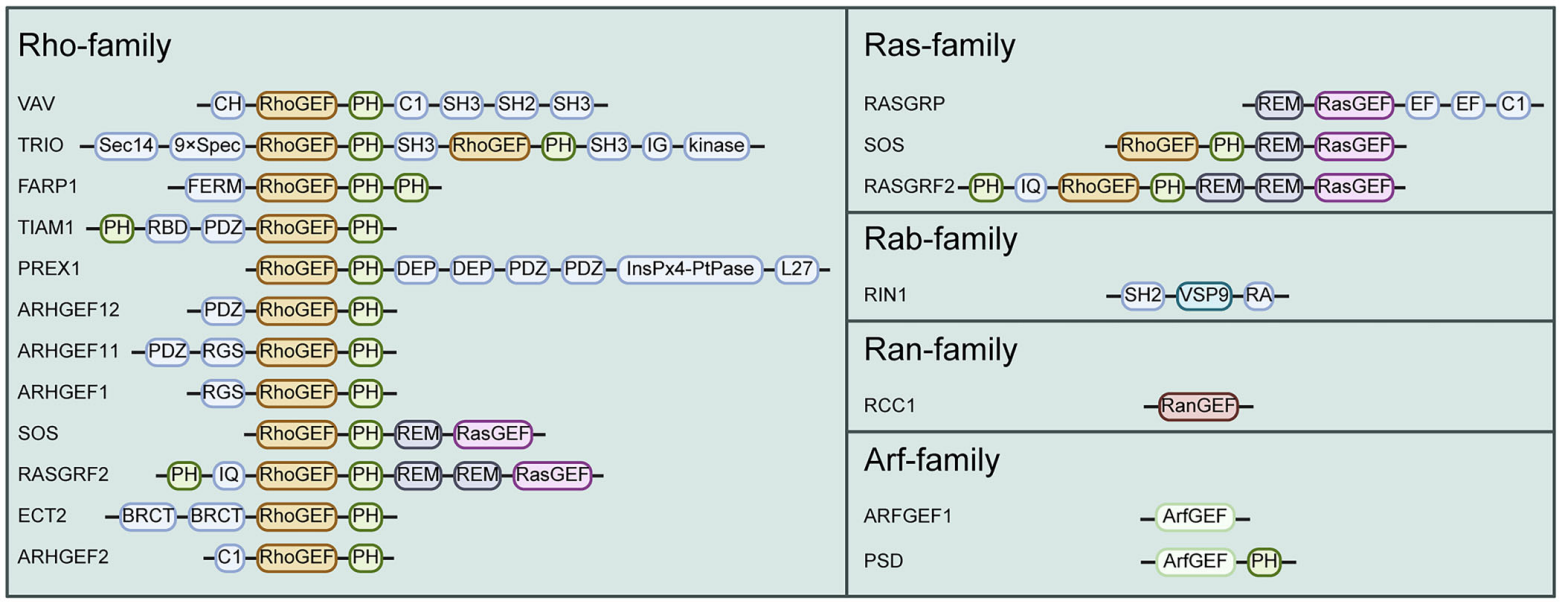
Analysis of the composition and functional characteristics of key protein domains in the GEFs. This figure illustrates the domain composition of members from different GEF families, including the Rho, Ras, Rab, Ran, and Arf families. Members of the Rho family GEFs (e.g., VAV, TRIO, TIAM1) typically contain the DH–PH domain (labeled as RhoGEF) and additional regulatory domains (e.g., SH3, SH2). Ras family GEFs (e.g., SOS, RASGRP) possess REM and CDC25 domains (marked as RasGEF). GEFs of the Rab, Ran, and Arf families exert their functions through characteristic domains such as VPS9, β‐helical repeat, and Sec7, respectively. Domain abbreviations: CH (calponin homology domain), SH3/SH2 (Src homology domain 3/2), DEP (dishevelled/Egl‐10/pleckstrin domain), REM (Ras exchange motif), BRCT (BRCA1 C‐terminal domain). This figure elucidates the molecular mechanisms by which the GEFs regulates the activities of various G proteins via specific domain combinations. Figures created with BioRender.com.

From the perspective of molecular classification and functional characteristics, the GEFs can be divided into multiple functional subclasses based on substrate specificity. RasGEFs (e.g., Sos1/2, RasGRF1/2, and RasGRP1‐4) primarily regulate the MAPK signaling pathway and cell proliferation/differentiation, with their mutations linked to Noonan syndrome and leukemia [7, 8, 9]. RhoGEFs, the largest subfamily (approximately 82 members in humans), include the Dbl family (e.g., Vav1–3, Tiam1) and dedicator of cytokinesis (DOCK) family, which participate in cytoskeletal dynamics and immune responses through characteristic DH–PH domains [10, 11, 12, 13] (Table 1). RabGEFs (e.g., DENND family, transport protein particle (TRAPP) complex, and vacuolar protein sorting 9 [VPS9] domain‐containing proteins) precisely regulate membrane trafficking processes, corresponding to over 60 Rab proteins [15, 16]. ArfGEFs (e.g., GBF1, ARNO) contain conserved Sec7 catalytic domains and are involved in Golgi function and vesicular transport [17, 18, 19, 20]. Additionally, there are specialized members such as the nuclear‐localized RanGEF and endoplasmic reticulum (ER)‐specific Sar1–GEF (Sec12) [21, 22, 23] (Table 1). These GEF subclasses typically exhibit modular structural features, consisting of catalytic domains and regulatory domains (e.g., PH, SH3), and achieve precise spatiotemporal activity regulation through phosphorylation modifications, subcellular localization control, protein–protein interactions (PPIs), and autoinhibitory mechanisms (Figure 2).

Beyond their functional diversity, the GEF family also exhibits remarkable evolutionary conservation. From an evolutionary standpoint, the GEFs demonstrates remarkable coevolution with small GTPase families. Small GTPases can be classified into five major families based on sequence and function: the highly conserved Ras family (regulating cell proliferation), the widely distributed Rho family (involved in cytoskeletal regulation), the Rab family (mediating membrane trafficking), the conserved Arf family (regulating vesicle formation), and the Ran family (controlling nucleocytoplasmic transport). Correspondingly, the GEFs has evolved matching subclasses, including the evolutionarily conserved Sos family (RasGEFs) from lower to higher organisms, the vertebrate‐specific RasGRF/GRP family, the Dbl family (RhoGEFs) with approximately 70 members in humans, the metazoan‐specific DENND family (RabGEFs), and the conserved Sec7 domain‐containing protein family (ArfGEFs). This coevolution primarily occurs through vertebrate whole‐genome duplication events leading to gene family expansion, functional divergence of duplicated genes (e.g., acquisition of immune regulatory functions by the mammalian Vav family), and domain evolution (with catalytic core domains being highly conserved while regulatory domains showing species‐specific variations) [14] (Table 1).

In terms of pathophysiological significance, GEF dysfunction is closely associated with various human diseases. Currently, at least 23 GEFs have been identified as oncogenes (e.g., ECT2, PREX1), whose hyperactivation or aberrant expression can cause persistent activation of downstream signaling pathways [4]. In the immune system, defects in GEFs (e.g., Vav1, DOCK8) lead to immune cell dysfunction, resulting in immunodeficiency or autoimmune diseases (e.g., DOCK8 deficiency syndrome) [14, 28]. Specific GEFs in the nervous system (e.g., Kalirin) are critically involved in neuronal development and synaptic plasticity, and their dysfunction may contribute to neuropsychiatric disorders such as schizophrenia [29]. These findings provide a new idea for the targeted treatment of related diseases. Currently, GEF inhibitors targeting Sos1 and GEF–H1 have entered clinical trials, and allosteric inhibitor strategies have also shown good application prospects.

Within the Rho GEF subfamily, ARHGEF2‐encoded GEF–H1 represents one of the most extensively studied members. Phylogenetic analysis reveals that GEF–H1 shares significant structural homology with ARHGEF18, ARHGEF28, and AKAP13, all containing highly conserved protein kinase C (PKC) C1 domains [2]. The discovery of GEF–H1 dates back to 1995 when Whitehead et al. identified its mouse homolog Lfc through screening for proteins capable of transforming mouse fibroblasts, with both sharing 88% amino acid sequence identity [30, 31, 32]. Notably, the first mammalian Rho GEF (Dbl) was initially discovered in diffuse B‐cell lymphoma, and its conserved DH domain serves as the characteristic catalytic module for approximately 70 RhoGEFs (including GEF–H1) [1, 2, 33, 34].

The molecular architecture of GEF–H1 exhibits multidomain cooperative regulation. The DH domain serves as the catalytic core, adopting an α‐helical conformation to bind Rho GTPase and promote its conformational change, showing higher affinity for nucleotide‐free RhoA and specifically facilitating GDP–GTP exchange [35]. The C1 domain, located at the N‐terminus of the DH domain, is a cysteine‐rich zinc finger motif structurally resembling the C1 diacylglycerol‐binding domain of PKC family members, which regulates GEF–H1 catalytic activity by mediating microtubule association through conserved cysteine residues [36]. The PH domain, positioned at the C‐terminus of the DH domain and originally identified in pleckstrin, possesses lipid‐binding capacity (e.g., phosphoinositides) and participates in membrane targeting and microtubule interaction, while being essential for nucleotide exchange activity by stabilizing the active conformation of the DH domain [37, 38]. The CC domain (coiled‐coil domain) at the C‐terminus of the PH domain forms an α‐helical coiled‐coil structure that mediates protein oligomerization and interactions (e.g., with gephyrin) [24, 39, 40]. The 14‐3‐3 binding site at the C‐terminus of the CC domain contains Ser886, which can be phosphorylated by various kinases (including Aurora A, PAK1, and CDK1), leading to microtubule association and subsequent inhibition of catalytic activity [41, 42, 43, 44]. Additionally, MARK3‐mediated phosphorylation of Ser151 generates an alternative 14‐3‐3 binding motif, further expanding regulatory complexity [45, 46].

With continuous advancements in research technologies, investigators employing comparative genomics, phylogenetic analysis, cryo‐EM structure determination, fluorescence resonance energy transfer (FRET) real‐time monitoring, and CRISPR screening have gained deeper insights into the GEF–GTPase system. These studies have not only elucidated the evolutionary history and regulatory mechanisms of the GEFs, revealing fundamental principles of cellular signal transduction, but also provided novel target perspectives for precision medicine. Future innovations, particularly in single‐cell technologies and AI‐assisted drug design, coupled with accumulating genomic data, will undoubtedly deepen our understanding of this crucial regulatory system and potentially lead to breakthroughs in disease diagnosis and treatment.

## 3 Core Regulatory Mechanisms of GEF–GTPase Systems

The activities of GEFs and their small GTPase substrates are tightly regulated through multiple mechanisms to ensure precise spatiotemporal control of cellular signaling. Structurally, GEFs catalyze the transition of GTPases from an inactive GDP‐bound state to an active GTP‐bound state by inducing conformational changes, with distinct families employing specialized domains such as DH–PH (RhoGEFs), REM–Cdc25 (RasGEFs), and Sec7 (ArfGEFs). Spatiotemporal regulation is achieved through subcellular localization, particularly via interactions with microtubules and membranes. For example, GEF–H1's activity is inhibited when bound to microtubules but activated upon their depolymerization, while membrane targeting domains like PH ensure proximity to GTPase substrates. Additionally, posttranslational modifications such as phosphorylation, ubiquitination, and SUMOylation dynamically modulate GEF function; phosphorylation of GEF–H1 at sites like Ser886 regulates its interaction with inhibitory 14‐3‐3 proteins, and ubiquitination regulates protein stability. These layered regulatory mechanisms enable rapid cellular responses to environmental cues, and their dysregulation is linked to pathologies such as cancer, neurodegeneration, and immune disorders.

### 3.1 Structural Basis of Nucleotide Exchange

The structural mechanism by which GEFs catalyze the transition of small GTPases from GDP‐bound to GTP‐bound states involves specific molecular interactions between conserved catalytic domains of GEFs and their cognate small GTPases. This process is elucidated by multiple high‐resolution structural studies [47]. Different classes of GEFs employ distinct structural strategies to accomplish this catalytic process.

For RhoGEFs (e.g., Dbl family members), the catalytic core consists of DH and PH domains. Crystallographic studies demonstrate that the DH domain binds to the Switch II region of Rho family GTPases (e.g., RhoA) through conserved hydrophobic residues (e.g., Leu697 and Phe698 in Dbl), inducing significant conformational changes [48]. This interaction disrupts the coordination network between the β‐phosphate group of GDP and Mg^2^⁺ ions. Simultaneously, the PH domain facilitates catalysis through two mechanisms: first, its β‐sheet structure stabilizes the active conformation of the DH domain [49]; second, it targets the complex to specific membrane compartments by binding to membrane phospholipids (e.g., PIP3) or protein interaction partners (e.g., ILK) [50].

RasGEFs (e.g., Sos1) activate Ras through their catalytic module (comprising the REM and Cdc25 domains). The helical hairpin (αH–αI) of the Cdc25 domain inserts into the nucleotide‐binding pocket of Ras, destabilizing its conformation [51]. The REM domain interacts with the Switch I region of Ras, inducing conformational changes that disrupt the P‐loop and impair the interaction between Lys16 and GDP, while the methyl side chain of Ala‐59 in Switch II occupies the Mg^2^⁺ binding site. Furthermore, Ras–GTP binding to the allosteric site of Sos1 induces a ∼10° rotation of the REM domain, stabilizing the active conformation of Sos1 and significantly enhancing the nucleotide exchange rate [50].

The catalytic mechanism of ArfGEFs (e.g., Arno) involves the hydrophobic groove (containing Phe151, etc.) of the Sec7 domain binding to the Switch I/II regions of Arf1, inducing conformational changes that disrupt GDP binding [52, 53]. The essential residue Glu156 is critical for activity, as its mutation completely abolishes nucleotide exchange. Membrane association is essential for this process: Arf1 must be anchored to membranes via N‐terminal myristoylation, whereupon its N‐terminal helix (residues 2–17) dissociates from the protein core and inserts into the lipid bilayer, synergistically cooperating with the Sec7 domain to facilitate GDP release [54, 55].

From the perspective of structural dynamics, crystallographic studies have revealed that GEF binding can induce significant conformational changes in the Switch regions of small GTPases. Taking the RhoA–LARG complex as an example, the α1 helix of the DH domain and its N‐terminal extension (αN1/αN2) directly interact with the Switch I region (e.g., Tyr34), while cooperatively engaging with the PH domain to mediate interactions with the Switch II region (e.g., Glu97 and Arg68), collectively destabilizing the GDP‐binding site [48]. Molecular dynamics simulations show that GEFs reduce the reaction energy barrier by inducing conformational changes in the GTPase active center (e.g., eclipsed conformation of γ‐phosphate oxygen atoms). Additionally, they promote charge redistribution, increasing the positive charge on the γ‐phosphorus atom [56]. Residence time measurements show that under (regulator of chromosome condensation 1) RCC1 catalysis, the GDP dissociation rate of the Ran–GTP complex increases from 1.5 × 10^−5^ to 21 s^−1^, with a half‐life reaching the millisecond timescale [57].

Among these GEFs, GEF–H1 (ARHGEF2) represents an important Rho GEF whose catalytic activity is closely tied to its structural features. Like typical RhoGEFs, GEF–H1 contains multiple functional domains including N‐terminal, C‐terminal, and PH domains that collectively mediate its interactions with microtubules and RhoA. Notably, the C‐terminal region of GEF–H1 functions as an autoinhibitory domain that suppresses GEF activity through intramolecular interactions with the catalytic core. Release from microtubules or phosphorylation‐induced relief of autoinhibition exposes the catalytic core, enabling GEF–H1 to promote GDP/GTP exchange on RhoA and activate downstream signaling pathways [58, 59]. Collectively, this regulatory mechanism exemplifies the structural and functional diversity of GEFs and their capacity for precise spatiotemporal regulation.

### 3.2 Spatiotemporal Control via Microtubules and Membranes

The catalytic activity of GEFs is tightly regulated through subcellular localization, a spatiotemporal control mechanism that has been demonstrated by multiple studies to ensure the specificity and accuracy of cellular signaling pathways [47]. Diverse molecular mechanisms collectively participate in regulating the localization and activation state of GEFs within cells, forming a highly intricate regulatory network.

In microtubule‐dependent regulation, GEF–H1 represents a classical example whose activity is tightly regulated by microtubule dynamics. Krendel et al. [60] confirmed through coimmunoprecipitation that GEF–H1 directly interacts with β‐tubulin via its N‐terminal hydrophobic region (1–190 aa), which suppresses its catalytic activity. As dynamic polymers of α/β‐tubulin heterodimers, microtubules critically determine GEF–H1's activation state: upon microtubule depolymerization, exposed α/β interfaces may induce conformational changes in GEF–H1, triggering its release and subsequent activation of the RhoA signaling pathway [61]. GEF–H1's dynamic regulation has been visualized in real time using FRET and linked to cell division and migration [62, 63]. GEF–H1 associates with microtubules through two distinct mechanisms—direct binding via its N‐terminal zinc finger and C‐terminal coiled‐coil domains [41, 60], and indirect recruitment via microtubule‐associated proteins (MAPs) [60]. Notably, FAM123A engages microtubule ends through its SKIP motif (residues 487–490), and its loss reduces microtubule polymerization rates while constitutively activating GEF–H1 [63, 64].

In the temporal dimension, the regulation of GEF–H1 exhibits complex kinetic characteristics. The most representative example is the LPS‐induced positive feedback loop: microtubule depolymerization → GEF–H1 release → Rho kinase activation → further microtubule depolymerization → additional GEF–H1 release, forming a “release storm” [65]. Notably, this cascade can be mitigated by antioxidants or microtubule‐stabilizing agents (e.g., Taxol), reflecting the plasticity of spatiotemporal regulation. Furthermore, the enhanced GEF–H1 activity resulting from EB1 depletion, as well as the mechanism by which lysophosphatidic acid (LPA) or thrombin activates GEF–H1 through G protein‐coupled receptors (GPCRs) signaling (independent of microtubule depolymerization), both highlight the coordinated regulation of GEF–H1 activity by microtubule dynamics and G protein signaling across temporal and spatial scales [66, 67, 68]. This precise combinatorial regulation enables GEF–H1 to sense diverse stimuli, including mechanical stress, reactive oxygen species (ROS), and bacterial infections, which trigger GEF–H1 release by disrupting microtubule structure [65, 69, 70].

Significant progress has also been made in the study of membrane localization mechanisms. Spatial positioning plays an equally critical role in membrane‐associated regulation. Multiple studies have demonstrated that PH domains mediate protein membrane localization by specifically recognizing phosphoinositides such as PtdIns (3,4,5)P_3_ or PtdIns (4,5)P_2_, while GEF–H1 binds directly to microtubules via its N‐terminal C1 domain [71, 72]. Furthermore, the microtubule‐binding ability of GEF–H1 is regulated by Par1b/MARK2‐mediated phosphorylation, which triggers its release from microtubules, thereby influencing its spatial distribution and function [72]. LPA or thrombin activates Gα and Gβγ subunits via GPCRs, potentially modulating microtubule dynamics or indirectly affecting GEF–H1 phosphorylation status to achieve its spatial redistribution [73]. This mechanism operates independently of microtubule depolymerization, highlighting the coordinated regulation between membrane signaling and the microtubule system. Moreover, the activation of GEF–H1 by mechanical stress or hyperosmotic pressure suggests that cells may sense mechanical stimuli through the membrane–cytoskeleton interface [74]. Surface plasmon resonance (SPR) measurements have determined that the typical PH domain binds PIP3 with an affinity (*K*_d_) in the range of 1–10 µm [75]. Immunogold‐labeled electron microscopy has confirmed that Arf–GEF ARNO localizes to the Golgi membrane via its PH domain interaction with PIP2, a specific positioning essential for Arf protein activation [76, 77, 78].

Research on compartmentalized signaling mechanisms has made a series of important advances. The Horiuchi team first revealed through in vitro reconstruction experiments that Rabex‐5, a GEF for Rab5, forms a functionally coupled complex with the effector protein Rabaptin‐5: Rabex‐5 catalyzes the GDP/GTP exchange of Rab5, while Rabaptin‐5 stabilizes its active state, together regulating endosomal membrane fusion [79]. This finding provides a paradigm for GEF‐effector synergistic action. Muller et al. systematically elucidated the compartmentalized regulation mechanism of Rho GTPases, discovering that Rho GEF/Rho GAP restricts signals spatially and cross‐regulates through subcellular localization (e.g., at focal adhesions), autoinhibition, and multiprotein complexes (such as PLEKHG4B–ARHGEF11/12), and also unraveling the crucial role of Rho GAP's low substrate specificity in maintaining activity gradients [80].

The complex localization regulatory mechanisms work together to guarantee the precise spatiotemporal control of GEF activity. Microtubule‐targeting agents can modulate this process through different mechanisms. Microtubule‐depolymerizing agents like plinabulin induce microtubule depolymerization by binding to the site between the α‐ and β‐tubulin subunits [61, 81]. In contrast, stabilizers such as Taxol analogs maintain microtubule stability by promoting lateral contacts between protofilaments [82, 83, 84]. Aberrations in any regulatory step may lead to diseases, for instance, DOCK8 mutations have been proven to cause immunodeficiency syndromes [85]. These discoveries underpin the development of microtubule‐targeted therapeutic strategies. By finely regulating microtubule dynamics or interfering with membrane‐associated signaling, it might be possible to achieve precise spatiotemporal control of GEF–H1 activity. However, fully comprehending the spatiotemporal characteristics of this intricate regulatory network still requires further research into the synergistic interactions between microtubules and membrane systems across different cellular contexts.

### 3.3 Posttranslational Modifications and Degradation

The activity and stability of GEFs are precisely regulated by various posttranslational modifications. Together, these modifications form a dynamic regulatory network, ensuring the accuracy and plasticity of cell signaling. Regulatory mechanisms include phosphorylation, ubiquitination‐mediated degradation, allosteric regulation, SUMOylation, and so on. The molecular mechanisms of these modifications have been confirmed by multiple high‐quality studies.

Phosphorylation has two distinct modes of action. Biochemical studies demonstrate that the Tyr174 site of the Vav1 protein can be specifically phosphorylated by Src family kinases. In vitro kinase reactions combined with mass spectrometry identified the exact phosphorylation site. Structural analysis by NMR revealed that phosphorylation at Tyr174 in the N‐terminal acidic domain (Ac, residues 132–176) of Vav1 induces a conformational change in the N‐terminal extension (residues 170–177), relieving the autoinhibition of the DH domain and activating Vav1's GEF activity to expose the GTPase binding site [86]. In contrast, during mitosis, Aurora A kinase phosphorylates GEF–H1 at Ser885. Immunoprecipitation shows Aurora A interacts with GEF–H1 in cells. Phosphorylation at Ser885 enables GEF–H1 to bind 14‐3‐3 protein, inhibiting GEF–H1's catalytic activity and preventing RhoA activation. SPR experiments show that phosphorylated GEF–H1 has a binding affinity (*K*_d_ = 0.21 ± 0.03 µm) for 14‐3‐3 protein nearly 50 times higher than the unphosphorylated form (*K*_d_ > 10 µm) [41].

The ubiquitination‐dependent degradation of Cdc25A has been well‐characterized. Here, the SCFβ–TrCP ubiquitin ligase plays a key role [87]. When the Ser76 site of Cdc25A is phosphorylated by Chk1 kinase, SCFβ–TrCP can recognize this modification. Also, the nontypical phosphodegron formed by the Ser79 and Ser82 sites of Cdc25A is identifiable by SCFβ–TrCP. These phosphorylation events act as degradation signals. Subsequently, Cdc25A is degraded through the ubiquitination and proteasome pathway. This process is crucial for cell‐cycle regulation. By rapidly reducing Cdc25A levels, it inhibits CDK activity, aiding cells in coping with DNA damage and other stress [88, 89]. Under nutrient stress, p62/SQSTM1 serves as a scaffold protein to mediate the degradation of GEFs (e.g., PREX1). Immunoprecipitation and confocal microscopy show that PREX1 and other GEFs bind to autophagosomes via the LC3‐interacting region (LIR, with a core sequence of [W/F/Y]XX[L/I/V]) and are eventually degraded by lysosomes [90].

Recent structural studies have advanced our understanding of allosteric regulation and autoinhibition mechanisms in GEFs. X‐ray crystallography has been used to determine the autoinhibited conformation of Sos1 at a resolution of approximately 3.6 Å. It has been revealed that the REM domain (residues 198–225) of Sos1 interacts with the Cdc25 catalytic domain (residues 564–1049) and works together with the DH–PH unit to shield the catalytic site, thereby achieving autoinhibition, rather than simply blocking it through a steric hindrance effect alone [91]. Nuclear magnetic resonance and FRET studies have shown that, in its resting state, Tiam1 forms an autoinhibited conformation through the binding of its PDZ ligand (residues 390–395) to the PH domain (residues 1012–1115). During cell migration, this interaction is disrupted by local PIP3 signaling, causing the dissociation constant (*K*_d_) to decrease from approximately 10 nM in the resting state to around 1 µm in the activated state [92].

SUMOylation plays a crucial role in regulating RanGEF RCC1. Research shows that RCC1's chromatin binding and dissociation are regulated by SUMOylation. In vitro studies indicate that SUMO E1 and E2 enzymes promote RCC1 and Ran dissociation from chromatin [93]. In yeast and mammalian cells, RanGAP (Rna1p/RanGAP1) is targeted to the nuclear pore complex (NPC) via SUMOylation, mediated by Nup358 in mammals [94]. In fission yeast, Nup132 absence releases Ulp1 (a SUMO‐specific protease) from the NPC, affecting Top2 and Pim1 (RCC1 homologs) SUMOylation levels [95]. These modification changes are vital for chromatin‐binding proteins during meiosis.

There are complex interplays among these posttranslational modifications. Immunoprecipitation and protein stability assays show that GEF–H1 phosphorylation can enhance its binding to the E3 ligase FBXO45, accelerating ubiquitination and degradation [96]. Also, quantitative MS (e.g., SILAC) analysis of HeLa cells in mitosis reveals that many regulatory proteins, including GEF–H1, undergo dynamic cell cycle‐dependent phosphorylation [97]. The mechanism of GEF–H1 stability regulation by a phosphorylation‐ubiquitination cascade indicates that this multilevel posttranslational modification network enables GEF activity to rapidly and accurately respond to intracellular and extracellular signals, such as cell cycle progression.

Among GEFs, the regulation of GEF–H1 activity is particularly complex, primarily through a posttranslational modification network, with phosphorylation playing a central role. To date, 36 phosphorylation sites have been identified on GEF–H1, mainly enriched in its C‐terminal autoinhibitory domain, forming a precise molecular switch system [42, 98]. Based on their functional characteristics, these phosphorylation sites can be clearly classified into two categories: activating and inhibitory.

Among the activating phosphorylation sites, Ser151, Ser645, and Thr678 are the most extensively studied. Phosphorylation at Ser151 is catalyzed by the LKB1–MARK3 kinase cascade, and the resulting phosphorylated site can specifically bind to 14‐3‐3 proteins, thereby releasing GEF–H1 from microtubules [45]. This mechanism plays a crucial role in the establishment of cell polarity. Ser645 is a specific target of Hormone Upregulated Neu‐associated Kinase (HUNK), and its phosphorylation activates the RhoA–LIMK1–cofilin axis, inhibiting epithelial–mesenchymal transition (EMT) by stabilizing F‐actin [99]. The regulation of Thr678 is unique, as while most phosphorylation events suppress GEF–H1 activity, phosphorylation at Thr678 mediated by ERK1/2 significantly enhances its activity. TNF‐α increases Thr678 phosphorylation by activating the ERK1/2 signaling pathway. Notably, when treating the Thr678A mutant with the ERK inhibitor PD98059, an abnormal increase in GEF–H1 activity was observed, implying the existence of other undiscovered activating sites [100, 101].

Inhibitory phosphorylation sites primarily activate GEF–H1 function through dephosphorylation mechanisms. The Ser886 site (Ser885 in mice) can be phosphorylated by various kinases, including PKA and PAK, promoting 14‐3‐3 protein binding and maintaining GEF–H1 in an inactive state [63, 68]. Gα and Gβγ subunits generated from GPCR signaling can competitively disrupt the GEF–H1–Tctex1–DIC complex, releasing GEF–H1 from microtubules but not fully activating it. Complete activation requires PP2A‐mediated dephosphorylation of Ser886 [66, 67]. Notably, profilin deficiency enhances Ser886 phosphorylation and activates the ROCK–myosin pathway, though the molecular mechanism remains unclear [102, 103]. The Ser959 site integrates signals from multiple kinases. Under growth stimulation, ERK phosphorylates this site to inhibit GEF–H1, while other kinases like MARK2 and Aurora B also target it [41, 42, 104].

The regulation of GEF–H1 phosphorylation is extremely complex. The same kinase can produce opposing effects via different sites: for example, ERK1/2 can both activate GEF–H1 through Thr678 and inhibit it through Ser959 [101, 105]. PAR1b kinase can synergistically regulate GEF–H1's subcellular localization and catalytic activity at multiple sites, including the N terminus (Ser143/172/186) and C terminus (Ser886/959) [72]. Additionally, a large regulatory network composed of kinases such as calmodulin‐dependent protein kinase I (Thr103), CDK5 (Ser122), PAK4 (Ser143), and Src (Tyr198), enables GEF–H1 to integrate diverse cellular signals [106, 107, 108, 109].

In addition to phosphorylation, GEF–H1 may also be regulated by other posttranslational modifications such as ubiquitination, but the exact functions and mechanisms of these modifications remain unclear. In terms of protein stability, preliminary evidence suggests that GEF–H1 may be degraded via the proteasomal or lysosomal pathway. However, the key information related to this process, including the E3 ligases, deubiquitinating enzymes, and degradation‐triggering signals, has yet to be discovered. It is worth noting that the activity of GEF–H1 can also be regulated by physical and chemical factors such as high osmotic pressure and mechanical stress [110, 111]. Nevertheless, how these stimuli are translated into specific posttranslational modification events requires further exploration.

Overall, GEF–H1 integrates various posttranslational modifications to form a multidimensional regulatory system, enabling it to respond precisely to different cellular signals. Future research should explore the cross‐regulatory relationships between these modifications and unveil their dynamic changes under physiological and pathological conditions. This will lay a theoretical foundation for developing GEF–H1‐targeted therapeutic strategies.

## 4 Physiological Functions of GEF–GTPase Networks

**FIGURE 3:**
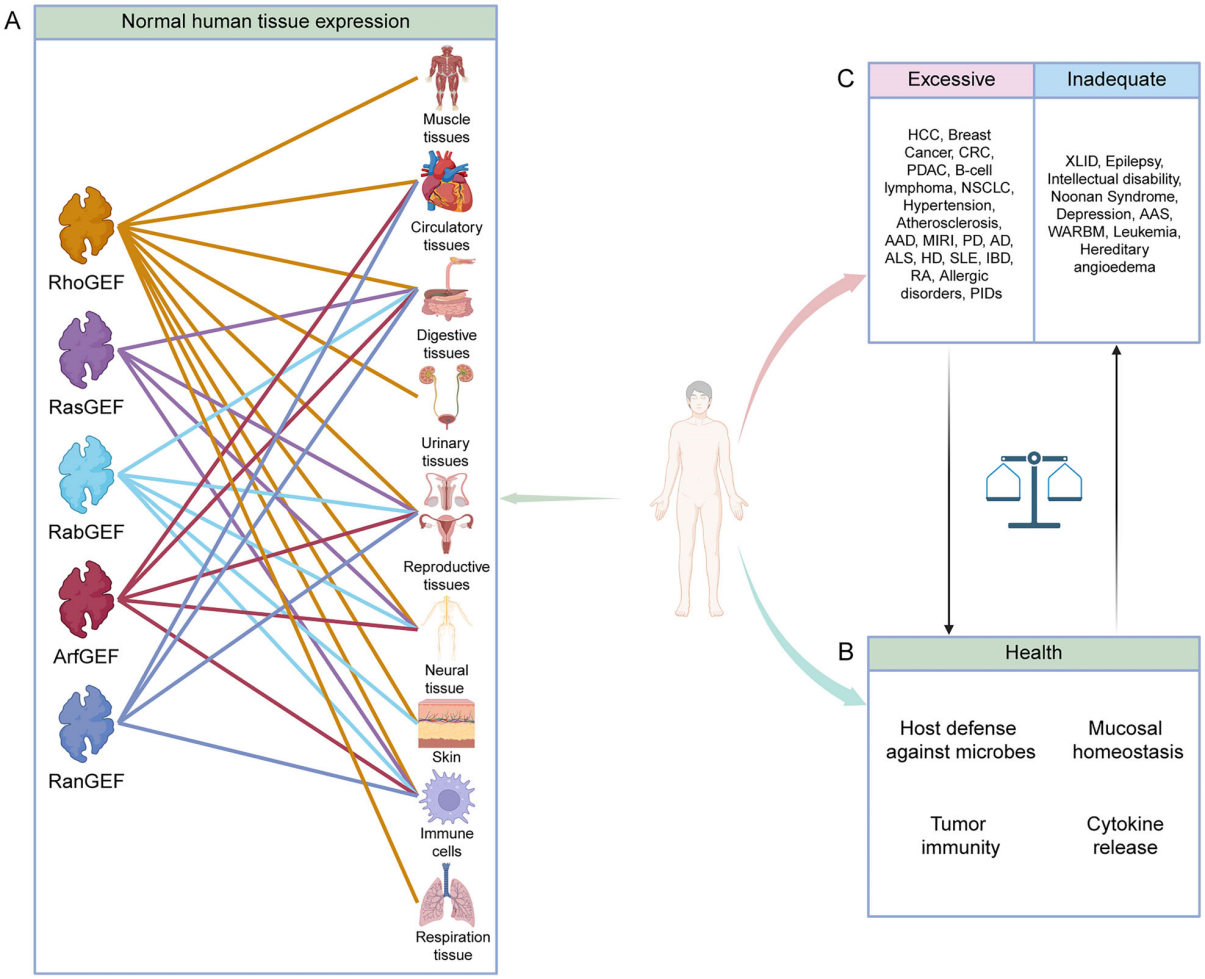
Expression patterns and pathological implications of GEF proteins in human tissues. This figure illustrates the expression patterns of RhoGEF, RasGEF, RabGEF, ArfGEF, and RanGEF in normal human tissues (A), their roles in various diseases caused by excessive or insufficient activity (C), and their critical functions in maintaining health, such as host defense against microbial infections, mucosal homeostasis, tumor immunity, and cytokine regulation (B). The affected tissues encompass multiple systems, including mucosal, circulatory, digestive, urinary, reproductive, neural, integumentary, immune, and respiratory tissues. Dysregulation of these GEFs has been implicated in a broad spectrum of diseases, including cancers (e.g., HCC, breast cancer), cardiovascular disorders (e.g., hypertension, atherosclerosis), neurodegenerative diseases (e.g., Parkinson's disease [PD], Alzheimer's disease [AD]), and immune‐related conditions (e.g., systemic lupus erythematosus [SLE], inflammatory bowel disease [IBD]). (*Note*: This figure is designed to mimic the structure and style of Figure 1 from the referenced article “Precise Orchestration of Gasdermins’ Pore‐Forming Function by Posttranslational Modifications in Health and Disease [112].” Figures created with BioRender.com.

GEFs can be classified into multiple subfamilies—including RhoGEF, RasGEF, RabGEF, ArfGEF, and RanGEF—based on their substrate specificity. These proteins are ubiquitously expressed in human tissues and regulate small GTPase activity, thereby maintaining essential physiological functions, including microbial defense and immune surveillance. Notably, abnormal expression or activity of GEFs is closely associated with numerous human diseases. Gain‐of‐function alterations in GEFs often promote pathological processes, including tumor progression, cardiovascular disorders, neurodegeneration, and autoimmune diseases, whereas loss‐of‐function mutations are associated with intellectual disability, epilepsy, and hematological malignancies. This subclassification underscores the pivotal role of GEFs in physiological homeostasis and disease pathogenesis, offering a molecular framework for developing therapies targeting specific GEF subfamilies (Figure 3). In the following sections, we elaborate on the mechanisms by which the GEF–GTPase axis regulates key physiological processes, such as cell motility, cytoskeletal dynamics, membrane trafficking, organelle homeostasis, immune responses, and barrier integrity.

### 4.1 Cell Motility and Cytoskeletal Dynamics

Cell motility and cytoskeletal dynamics are central to various physiological and pathological processes, primarily regulated by a complex network composed of Rho family GTPases (RhoA, Rac1, Cdc42) and their regulators (GEFs/GAPs) [113, 114, 115, 116]. This regulatory system coordinates critical biological behaviors such as cell migration, polarity establishment, and mechanical stress response through precise spatiotemporal control of GTPase activation patterns. Among these regulators, GEF–H1 serves as a pivotal molecule connecting microtubule dynamics with Rho signaling pathways, exhibiting unique regulatory mechanisms and diverse biological functions [24, 60].

GEF–H1 activity is strictly regulated by microtubule dynamics, making it a molecular sensor for cellular perception of microenvironmental changes [24, 60]. When microtubules are in a stable polymerized state, GEF–H1 remains inactive through binding to microtubules via its N‐terminal domain [41, 60]. Conversely, upon microtubule depolymerization or posttranslational modifications (e.g., HDAC6‐mediated deacetylation or αTAT1‐catalyzed acetylation), GEF–H1 is released and activates downstream Rho GTPases (including RhoA, Rac1, and Cdc42) [117]. This microtubule‐tension sensing mechanism enables cells to dynamically adjust cytoskeletal reorganization strategies in response to local microenvironmental changes [118]. For instance, at the migration front, localized microtubule depolymerization leads to regional GEF–H1 activation, coordinating migration through two parallel mechanisms: enhancing local contractility via the RhoA/ROCK/MLC pathway while promoting lamellipodia and filopodia extension through the Rac1/Cdc42–WAVE–Arp2/3 pathway [119, 120]. This dual regulation allows cells to flexibly adapt to extracellular matrices with varying physical properties.

During cell migration, GEF–H1 forms a sophisticated regulatory network with functional complementarity to other RhoGEFs. In mesenchymal migration, GEF–H1 exhibits significant synergy with p63RhoGEF (ARHGEF25) [114]. While GEF–H1 primarily drives lamellipodia formation and protrusion through Rac1/Cdc42 activation, p63RhoGEF maintains migration polarity and directional persistence via RhoA activity [119]. Studies show that p63RhoGEF inhibition results in multiple nonpolarized lamellipodia, a phenotype resembling GEF–H1 overexpression‐induced Rac1 hyperactivation, suggesting intricate feedback regulation [121]. Further research reveals that mixed lineage kinase 3 can inhibit p63RhoGEF's RhoA‐activating capacity through direct binding independent of its kinase activity, a process positively regulated by Gαq‐coupled receptor signaling and negatively modulated by JNK pathway feedback, forming a dynamically balanced regulatory circuit [122]. In amoeboid migration, GEF–H1 primarily forms a cascade with LARG (ARHGEF12): initial GEF–H1 activation enhances myosin contractility through RhoA–ROCK signaling, while diaphanous‐related formin (Dia1), as a classical RhoA effector, not only promotes linear actin polymerization but also relieves LARG autoinhibition, establishing a Dia1–LARG–RhoA positive feedback loop that amplifies contractile signals [123]. Additionally, RSK2 kinase significantly enhances LARG's GEF activity by phosphorylating Ser1288, a modification particularly critical for tumor cell bleb‐based invasion upon EGF or serum stimulation [124].

Mechanical signal integration and transduction represent one of GEF–H1's most prominent functional characteristics. When cells experience mechanical stimuli such as substrate stiffness changes or fluid shear stress, GEF–H1 participates in force signal transmission via the integrin–microtubule coupling pathway [118]. Mechanical tension first promotes microtubule network acetylation (catalyzed by αTAT1), leading to GEF–H1 release and local RhoA activation [125]. The activated RhoA–ROCK pathway enhances myosin contractility through MLC phosphorylation while stabilizing focal adhesions and promoting YAP nuclear translocation [126]. Nuclear YAP subsequently transcriptionally regulates GEF–H1 expression, forming a mechanical force–microtubule–GEF–H1–YAP positive feedback loop [127]. This mechanism exhibits spatiotemporal complementarity with Arhgef11/12‐mediated “Rac–Rho crosstalk”: when Rac activation drives edge protrusion, Arhgef11/12 are specifically recruited to the protrusion base to activate Rho for contraction, whereas GEF–H1 primarily regulates contraction duration and intensity by responding to microtubule deformation [128]. Experimental evidence shows that simultaneous knockdown of GEF–H1 and Arhgef11/12 abolishes the periodic contraction–expansion rhythm of cell migration, resulting in random movement patterns [129, 130].

GEF–H1's functional implementation also relies on crosstalk with multiple signaling pathways. The calcium signaling system forms a precise negative feedback network with GEF–H1: TRPC channel‐mediated Ca^2^⁺ influx activates Calpain protease to cleave microtubules, indirectly promoting GEF–H1 release; meanwhile, Ca^2^⁺‐dependent CamKII phosphorylates GEF–H1 at Ser885 to inhibit its activity [60, 130]. This “microtubule–Ca^2^⁺–GEF–H1” negative feedback constitutes a molecular clock controlling migration persistence. In cell polarity establishment, GEF–H1 interacts with the Par3–Par6–aPKC polarity complex to maintain anteroposterior axis stability by regulating Cdc42 activity, while the ELMO1–Dock180 complex locally activates Rac1 at the leading edge, ensuring directional migration precision [106, 131, 132, 133]. Within receptor tyrosine kinase (RTK) networks, Rac–GEFs such as FARP1, ARHGEF39, and TIAM2 (activated by EGFR/c‐MET in lung adenocarcinoma) activate Rac1 via PI3K to regulate distinct aspects of membrane ruffling dynamics (FARP1 and ARHGEF39 maintain ruffles stability, while TIAM2 participates in their formation) [134, 135]. GEF–H1 coordinates subsequent contraction events by responding to RTK‐induced microtubule remodeling, completing the migration cycle [99].

From a pathological perspective, dysregulated synergy between GEF–H1 and other GEFs drives multiple disease processes. During tumor metastasis, aberrant GEF–H1 release in breast cancer cells requires microtubule‐severing enzymes like FL2, with activation thresholds strictly regulated by scaffold proteins such as IQGAP1 [136, 137]. Simultaneously, BPGAP1 promotes invasive protrusion formation through its dual regulatory mechanism (C‐terminal RhoGAP activity suppresses RhoA, while the N‐terminal region scaffolds Vav1 to activate Rac1), synergizing with GEF–H1 to enhance metastatic potential [138, 139, 140]. Clinical data analysis reveals that high expression of both GEF–H1 and FARP1/ARHGEF39 significantly correlates with poor patient prognosis [8]. In immune cell migration, neutrophil Tiam1 regulates migration–adhesion balance by suppressing Rac1/β2 integrin signaling (Tiam1 deficiency enhances ICAM1 adhesion but impairs migration), while GEF–H1 controls chemokine‐induced polarity reorientation through microtubule dynamics [141, 142, 143]. During tissue repair and fibrosis, GEF–H1‐mediated YAP activation collaborates with ARHGEF17 (TEM4)‐driven Gβγ–RhoA signaling to promote fibroblast contraction and collagen deposition, emerging as novel therapeutic targets [144, 145].

Current targeted therapeutic strategies primarily focus on multipathway synergistic interventions. Examples include combined use of ROCK inhibitors (blocking GEF–H1/LARG downstream signaling) with microtubule stabilizers (inhibiting GEF–H1 release), or multitarget inhibitors simultaneously targeting the ARHGEF7–STIL–Rac1 axis and GEF–H1–RhoA axis [64, 99, 134, 146, 147]. Future research should address several key scientific questions: precise subcellular localization mechanisms of GEFs (e.g., GEF–H1 enrichment dynamics at microtubule plus‐ends, ARHGEF11/12 recruitment to contractile rings) [60, 148]; their dynamic regulatory networks in organoid migration and in vivo microenvironment adaptation; and developmental disease implications of different GEF mutation combinations. These investigations will not only deepen understanding of fundamental cell motility principles but also provide critical theoretical foundations for developing novel therapeutic strategies against tumor metastasis, immune disorders, and fibrotic diseases.

### 4.2 Membrane Trafficking and Organelle Homeostasis

The regulation of membrane trafficking and organelle homeostasis constitutes a complex and precise system involving the coordinated action of multiple GEF families. These proteins form the core regulatory network of intracellular trafficking pathways by spatiotemporally activating small GTPases (e.g., Rab, Arf, Rho) [15, 149]. The following sections will elaborate on the structural characteristics, functional mechanisms, and critical roles of various GEF families in physiological and pathological processes.

The TRAPP complex, as an important multisubunit GEF, exhibits remarkable functional diversity in eukaryotic cells. Based on subunit composition and functional differences, TRAPP complexes can be divided into two main subtypes: TRAPPII and TRAPPIII [150, 151]. TRAPPIII is evolutionarily more conserved, with its core composed of highly conserved subunits (e.g., TRAPPC1–4, 8, 11–13), which activate Rab1 to regulate the early secretory pathway from the ER to the Golgi apparatus, as well as autophagy [150, 152, 153]. Cryo‐EM structural studies have revealed the unique clamp‐like architecture of TRAPPIII: the core consists of multiple small subunits sandwiched between the two large subunits TRAPPC8 and TRAPPC11, with TRAPPC12 and TRAPPC13 located at the junction. This distinctive structure not only determines its substrate specificity but is also closely associated with disease‐related mutation sites, such as the Asp46 mutation in TRAPPC2, which causes spondyloepiphyseal dysplasia [153]. In contrast, the TRAPPII complex contains additional specific subunits, such as Trs120 and Trs130, built upon the TRAPPIII core, primarily activating Rab11 to regulate late Golgi trafficking and endosomal recycling. Structural studies indicate that TRAPPII forms “lid” and “leg” structures through Trs120 and Trs130, which not only occlude the active site but also elevate the distance between the active site and the membrane surface, thereby enabling specific activation of Rab11 [154]. In plant systems, TRAPPII exhibits unique regulatory mechanisms, such as integrating environmental signals through phosphorylation modifications of TRS120 to modulate adaptive growth decisions [155].

Although TMEM131 does not belong to the classical GEFs, it exhibits GEF‐like cooperative functions in membrane trafficking regulation. Studies have shown that TMEM131 plays a critical role in intracellular collagen assembly and secretion. Its N‐terminus contains a PapD‐like chaperone domain (PapD‐L) that specifically recognizes and recruits immature collagen monomers, promoting their proper folding and assembly, while its C‐terminus harbors a TRAPID domain that directly interacts with TRAPPC8, a key component of the TRAPPIII complex, driving directional collagen transport from the ER to the Golgi [156, 157]. This regulatory mechanism is highly conserved across multiple model organisms (including nematodes, fruit flies, and humans), underscoring its evolutionary importance. Loss of TMEM131 function leads to severe collagen secretion defects and triggers ER stress responses (e.g., the unfolded protein response), suggesting its pivotal role in maintaining ER homeostasis. Notably, the C‐terminal TRAPID domain of TMEM131 not only mediates binding to TRAPPC8 but also further modulates the transport efficiency of COPII vesicles, thereby influencing collagen secretion [158]. These findings provide new insights into the molecular mechanisms of extracellular matrix assembly and fibrotic diseases.

The BLOC‐3 complex, composed of two subunits (HPS1 and HPS4), is a specialized GEF that specifically activates Rab32 and Rab38 GTPases, playing a central role in the biogenesis and functional maintenance of lysosome‐related organelles (LROs) [159]. In melanocytes, BLOC‐3 regulates melanosome maturation through Rab32/38 activation, specifically by modulating the recycling of VAMP7: as a critical v‐SNARE protein, VAMP7 is initially transported to melanosomes via the BLOC‐1 complex for membrane fusion, followed by retrieval from melanosomes through a BLOC‐3–Rab38/VARP‐dependent pathway [160, 161]. BLOC‐3 dysfunction is closely linked to Hermansky–Pudlak syndrome (HPS), a genetic disorder characterized by oculocutaneous albinism, platelet dysfunction, and pulmonary fibrosis. HPS patients exhibit impaired Rab32/38 activation due to BLOC‐3 mutations, leading to functional abnormalities in LROs such as melanosomes and platelet dense granules [162, 163, 164]. Additionally, in host defense mechanisms, the BLOC‐3‐dependent Rab32 pathway transports antimicrobial molecules into pathogen‐containing vacuoles (e.g., Salmonella Typhi), while pathogens have evolved effector proteins like GtgE to cleave Rab32 and evade this defense mechanism. This discovery provides new insights into host‐pathogen interactions [165, 166, 167].

In the yeast secretory pathway, a sophisticated Rab GEF cascade exists: the upstream Rab GTPase Ypt32, in its GTP‐bound state, recruits Sec2 (the GEF for Sec4) to secretory vesicle surfaces, where Sec2 subsequently activates the downstream Rab GTPase Sec4, ultimately promoting targeted vesicle fusion with the plasma membrane [168, 169]. This cascade reveals the temporal regulation mechanism of Rab GTPases in membrane trafficking: Ypt32‐GTP → Sec2 recruitment → Sec4 activation → vesicle transport and fusion, ensuring the orderly progression of the secretory pathway. Similar regulatory mechanisms exist in higher eukaryotes. For instance, in mammals, DENND1A, as a specific GEF for Rab35, regulates endocytosis and receptor‐mediated trafficking by modulating Rab35 activity. Genetic variations in DENND1A are significantly associated with bovine ovarian morphology and function, such as ovarian width, corpus luteum diameter, and mature follicle count, highlighting the importance of RabGEFs in reproductive system development. Moreover, aberrant DENND1A expression is linked to the pathogenesis of polycystic ovary syndrome, providing new clues for understanding the molecular basis of this disease [170, 171].

The ARF–GEF proteins regulate COPI/COPII vesicle formation and trafficking by activating ADP‐ribosylation factor (Arf) GTPases. Different ARF–GEF members exhibit distinct subcellular localization and functional specificity: Golgi‐localized GBF1 is a key factor in maintaining Golgi structure and function. Studies show that treatment with GBF1‐specific inhibitor Golgicide A causes COPI dissociation from Golgi membranes, leading to Golgi and trans‐Golgi network disassembly, while blocking the secretion of soluble and membrane‐anchored proteins, trapping them in the ER–Golgi intermediate compartment (ERGIC). In contrast, the broad‐spectrum ArfGEF inhibitor Brefeldin A (BFA) affects both COPI localization and membrane binding of vesicle coat proteins (e.g., AP‐1, GGA3), demonstrating broader inhibitory effects [172, 173]. In plant cells, Arabidopsis ARF–GEFs (e.g., GNOM and GNL2) exhibit tissue‐specific functions. GNOM primarily mediates endosomal recycling, regulating the polar localization of auxin transporter PIN, while pollen‐specific GNL2 exclusively controls pollen germination and pollen tube growth, with its inhibition specifically blocking pollen tube tip growth [174, 175]. The small‐molecule inhibitor Endosidin 4 (ES4) targets multiple SEC7 domain‐containing ArfGEFs (including GNOM, BIG1, BIG4, and BIG5), broadly inhibiting membrane trafficking by interfering with ARF1 membrane‐binding activity. Unlike BFA, ES4 does not induce typical BFA body formation but affects membrane trafficking through a more refined mechanism [176].

Rho family GEFs play diverse roles in cell signaling transduction and organelle dynamics regulation. GEF–H1, as a critical regulator of the Rho family, activates multiple Rho GTPases (e.g., RhoA and RhoB) and regulates vesicle trafficking through various signaling pathways, playing an essential role in cellular vesicle transport [146]. When microtubules are intact, c‐Src vesicles primarily undergo long‐distance directional transport along microtubules. However, upon microtubule depolymerization, GEF–H1 is released from microtubules and activates Rho GTPases, thereby modulating vesicle transport through distinct mechanisms. On one hand, GEF–H1 binds to Sec5 in the vesicle complex, dependent on Ral GTPase regulation, activating RhoA, which in turn regulates vesicle complex assembly and positioning, promoting vesicle targeting and fusion [146]. On the other hand, GEF–H1 activates Rho GTPases to induce actin polymerization [177], forming actin comet tails that drive short‐distance, random vesicle movement [178]. Additionally, GEF–H1 can activate the RhoA–PLCε–DAG–PKD signaling pathway to promote the fission and transport of Rab6‐positive vesicles from the Golgi, ensuring accurate cargo delivery to the plasma membrane, particularly at focal adhesion sites. GEF–H1 not only responds to microtubule depolymerization but can also be activated via GPCR signaling pathways to further regulate vesicle transport and cell migration. These mechanisms play vital roles in cell division, migration, and secretion. GEF–H1 deficiency leads to vesicle accumulation and transport delays, while Ral GTPase enhances this process by strengthening the interaction between GEF–H1 and Sec5 [179]. Vav2, another important Rho GEF, influences neurotransmitter homeostasis by regulating the membrane expression of the dopamine transporter (DAT). Research indicates that glial cell line‐derived neurotrophic factor activates Vav2 through its receptor Ret, promoting DAT internalization from the plasma membrane and reducing dopamine reuptake. This process strictly depends on Vav2's GEF activity, with DAT and Ret receptors forming direct complexes on the plasma membrane, providing a structural basis for DAT membrane trafficking [180]. Another intriguing example is RIN1, which activates the GTPase activity of mitochondrial fusion protein Mfn2 through a noncanonical mechanism. Studies reveal that inactive Smad2 binds to Mfn2 in the cytoplasm and recruits the GEF protein RIN1 to form a trimeric complex that activates Mfn2, thereby promoting mitochondrial fusion. This process is entirely independent of the classical transcriptional function of TGF‐β/Smad2, unveiling a novel mechanism of GEF proteins in organelle dynamics regulation [181]. In disease‐related research, the “regulation of RhoA activity panel” (RRAP) has been identified as an important biomarker for gastric cancer prognosis. RRAP mutations reduce the activity of migration‐related pathways (e.g., adhesion junctions, cytoskeleton regulation) while altering the tumor immune microenvironment (e.g., increased CD8+ T cell infiltration), changes closely associated with membrane trafficking and organelle homeostasis [182].

Recent studies have uncovered functional synergy between P4–ATPase lipid flippases (e.g., Drs2) and multisubunit tethering complexes (MTCs, including TRAPPIII, GARP, and COG) [183, 184]. Drs2 specifically binds to the Trs85 subunit of TRAPPIII through its N‐terminal conserved I (S/R)TTK motif, an interaction critical for stabilizing TRAPPIII localization on Atg9 vesicle membranes, thereby regulating Atg9 transport from endosomal pools to the Golgi and influencing early stages of autophagy [185]. Notably, this interaction exhibits significant temperature dependence, being more pronounced under low‐temperature conditions. Similar regulatory mechanisms exist in other systems, such as the GARP complex modulating the recycling and function of Dnf1 and Dnf2 through comparable mechanisms. These findings collectively demonstrate that P4‐ATPases and MTCs form an intricate regulatory network central to maintaining membrane asymmetry, vesicle trafficking, and organelle homeostasis. This study employed innovative proximity imaging of complex territories and crosslinking mass spectrometry techniques, providing powerful tools for investigating dynamic changes in low‐abundance protein interactions within living cells [185].

In summary, GEF proteins maintain organelle homeostasis through complex regulatory networks, and their dysregulation contributes to the pathogenesis of various diseases. Future research should focus on the following directions: (1) elucidating the dynamic assembly and activation mechanisms of GEF complexes (2); investigating the precise regulation of GEF activity in different cellular contexts (3); developing small‐molecule tools specifically targeting GEFs; and (4) exploring GEF roles in novel membrane trafficking pathways. These advances will deepen our understanding of organelle homeostasis regulation and provide new strategies and targets for treating related diseases.

### 4.3 Immune Regulation and Barrier Integrity

The pathophysiological mechanisms underlying immune regulation and barrier integrity constitute a highly complex process involving intricate regulatory networks of multiple GEF proteins. These proteins regulate immune cell function and barrier homeostasis through spatiotemporal control of expression and activity.

In immune regulation, the KIR family of inhibitory receptors represents a crucial immune checkpoint system. KIR2DL5 and KIR3DL3 recognize the overexpressed immunoregulatory molecules PVR (CD155) and HHLA2 on tumor cell surfaces, forming immunosuppressive synaptic structures. Mechanistically, these receptors recruit SHP‐1 and SHP‐2 phosphatases via their intracellular ITIM motifs, exerting multilayered effects on downstream signaling: first, they directly inhibit Vav1 phosphorylation and activation; second, they block the ERK1/2/p90RSK signaling cascade; ultimately leading to downregulation of NF‐κB transcriptional activity [186, 187]. Notably, these ligands exhibit functional duality in the tumor microenvironment. PVR and HHLA2 bind both inhibitory (KIR2DL5, KIR3DL3, TIGIT) and activating (DNAM‐1) receptors, suppressing or stimulating immune responses. This signal‐balancing mechanism demands precise spatiotemporal targeting strategies [186, 187].

The VAV family proteins serve as central regulatory nodes in immune cell signal transduction. VAV1, as a crucial GEF molecule for Rho/Rac family, demonstrates multifaceted functions in T cell receptor signaling: structurally, it promotes Rac1 GDP/GTP exchange through its GEF‐dependent activity, driving cytoskeletal reorganization and immune synapse formation; in signal transduction, it acts as a scaffold protein participating in spatial organization of TCR signal complexes; functionally, it regulates T cell activation thresholds and effector differentiation [188]. Under pathological conditions, acquired VAV1 mutations promote lymphomagenesis through multiple mechanisms: enhancing GEF‐dependent RAC1 activation, altering signal transduction via GEF‐independent adaptor functions, and potentially losing tumor suppressor activity, ultimately leading to aberrant follicular helper T cell (Tfh) activation and malignant transformation [6]. Meanwhile, the VAV1–Themis functional complex is critical for maintaining T cell homeostasis. Recent studies show that Themis competitively inhibits SHP‐1 phosphatase‐mediated VAV1 dephosphorylation via its SH2 domain, sustaining VAV1 activation‐a positive feedback mechanism essential for proper T cell effector function [189]. In B cell development, VAV3 epigenetically regulates PRC1 complex chromatin localization and activity, finely tuning cell cycle checkpoint genes including Cdkn2a and Cdkn2b. VAV3 deficiency causes gene expression dysregulation, increasing B cell precursor apoptosis and proliferation arrest, providing new insights into B cell malignancy mechanisms.

DOCK family proteins exhibit remarkable functional diversity in immune regulation and barrier maintenance. DOCK2 activates the Rac–PAK1 axis via its unique DHR‐2 domain, regulating mast cell degranulation. Molecularly, DOCK2 forms dynamic complexes with MRGPRX2/B2 receptor downstream molecules, promoting Rac conformational changes through spatial rearrangement, ultimately leading to secretory apparatus assembly and activation [190]. In antifungal immunity, DOCK2 activity undergoes strict posttranslational regulation: fungal components activate SYK kinase via pattern recognition receptors, specifically phosphorylating DOCK2 on Y985 and Y1405. These phosphorylation events enhance DOCK2–Rac GTPase affinity and promote membrane localization, spatiotemporally controlling NADPH oxidase activation and ROS production [191]. DOCK8 deficiency causes combined immunodeficiency (CID) with complex pathological features: impaired immune cell chemotaxis and synapse formation at cellular level; disrupted WASp–Arp2/3‐mediated actin reorganization at molecular level; and Treg dysfunction, Th2 bias, and skin barrier defects at systemic level, clinically manifesting as eczema, recurrent infections, and severe allergies [192, 193]. Notably, while hematopoietic stem cell transplantation (HSCT) can restore immune function in DOCK8‐deficient patients, skin barrier repair often requires longer, suggesting DOCK8's autonomous role in epithelial maintenance [194].

In dynamic barrier maintenance, ARHGEF18 (p114RhoGEF) plays central regulatory roles. During epithelial collective migration, ARHGEF18 interacts with polarity complexes via its N‐terminal PDZ domain, spatially restricting RhoA activation to precisely regulate local actomyosin contractility‐providing migration force while maintaining E‐cadherin‐mediated junctions through mechanical tension feedback [195]. In vascular barrier regulation, TNF‐α‐induced disruption involves multilayer signaling: initial TNFR1–ASK1–MKK3/6–p38MAPK activation phosphorylates GEF–H1 at Ser885; phosphorylated GEF–H1 translocates from microtubules to membrane; ultimately activating local RhoA/Rac1 pathways, causing MLC phosphorylation, stress fiber formation, and tight junction protein internalization [196]. Recent studies show that garlic‐derived S‐1‐propenylcysteine (S1PC) protects endothelial barriers via dual mechanisms: competitively inhibiting p38MAPK‐mediated GEF–H1 phosphorylation and enhancing MLCP activity, showing therapeutic potential for inflammatory vascular leakage [196].

The TIAM1–RAC1 axis exhibits surprising pleiotropy in neuroimmune regulation and vascular barrier maintenance. In neuropathic pain, TIAM1–PSD‐95 complexes spatially restrict RAC1 activation to dendritic spines, promoting cofilin‐mediated actin reorganization and PAK1‐dependent GluN2B phosphorylation, ultimately causing central sensitization [197]. In aortic aneurysms, inflammatory RNS induce Septin2 S‐nitrosylation at Cys111, reducing Septin2–TIAM1 affinity and causing TIAM1 redistribution. Released TIAM1 hyperactivates RAC1–NF‐κB, promoting inflammatory cytokine production and ECM degradation via MMP9 [198]. RAC1 inhibitor NSC23766 or nitrosative stress scavengers show therapeutic efficacy in animal models.

SOS family proteins play complex roles in immune microenvironment regulation. Beyond canonical RASGEF function in KRAS‐mutant tumors, SOS1 exhibits unique pathology in BCR–ABL+ leukemia: PH domain‐mediated interaction with BCR–ABL fusion protein confers constitutive kinase activity, enhancing both RASGEF function and RAC1 activation via C‐terminal proline‐rich regions, promoting leukemic cell proliferation [199]. In NF1‐deficient tumors, SOS1‐mediated signal reactivation enables immune escape. NF1 loss causes sustained RAS–GTP levels, and while MEK inhibitors transiently block ERK signaling, SOS1‐dependent feedback drives resistance. MEK inhibitor/SOS1 degrader combinations enhance antitumor immunity [199, 200]. Environmental stressors like heat shock epigenetically regulate GEF proteins: heat‐induced METTL3 upregulation enhances SOS2 mRNA m6A modification and translation, activating NLRP3 inflammasome via ERK and compromising blood‐testis barrier integrity [201], providing new molecular insights into environmental effects on barrier function through GEF proteins.

## 5 Dysregulation in Human Diseases

Emerging evidence has shown that GEFs and their small GTPase substrates play key roles in various human diseases through complex regulatory mechanisms. In cancer, GEFs like the Vav family and GEF–H1 drive tumor initiation, progression, and drug resistance by abnormally activating Ras and Rho signaling. Specifically, Vav1 synergizes with KRAS mutations to promote pancreatic cancer, while GEF–H1 facilitates hepatocellular carcinoma (HCC) metastasis by modulating RhoA‐dependent cytoskeletal changes. Neurodegenerative diseases are marked by disrupted synaptic vesicle proteins such as Rab3 and Synaptotagmin (Syt), along with abnormal activation of RhoGEFs such as Ephexin5 (E5) and LRRK2. These aberrant activations impair neuronal structure and mitochondrial homeostasis. In cardiovascular diseases, RhoGEFs such as ARHGEF1 and LARG contribute to hypertension and aneurysm formation through activation of the Ang II‐induced RhoA/ROCK pathway, leading to endothelial dysfunction. Immunological disorders also arise from GEF mutations that disrupt lymphocyte signaling, including Vav1 mutations in T‐cell lymphomas, DOCK8 deficiency in hyper‐IgE syndrome, and GEF–H1‐driven Th17 polarization in lupus. These findings highlight the importance of GEF networks in disease mechanisms and suggest potential therapeutic targets, such as SOS1 inhibitors and RhoA modulators, for restoring dysregulated signaling pathways.

### 5.1 Cancer: From Oncogenic GEFs to Metastatic Reprogramming

**FIGURE 4:**
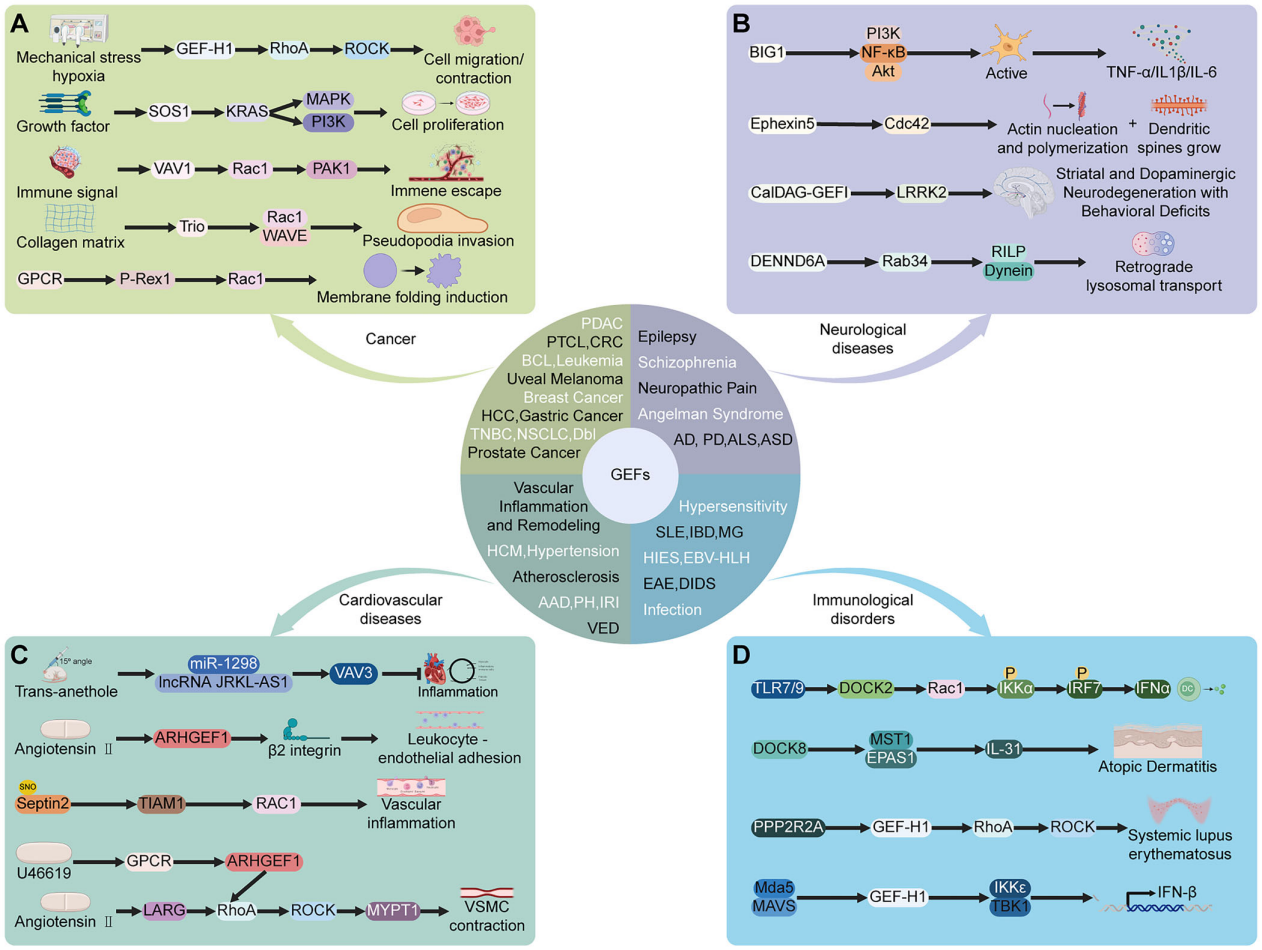
GEF‐mediated signaling networks in cellular processes and human diseases. This figure emphasizes the key role of GEFs in cellular signaling pathways and their links to human diseases. GEFs are involved in cancers (e.g., PDAC, leukemia), neurological disorders (e.g., AD, PD), autoimmune diseases (e.g., SLE, IBD), cardiovascular conditions, and immune‐related diseases. (A) In tumors, GEFs (e.g., GEF–H1, SOS1) activate Rho GTPases (e.g., RhoA, Rac1), regulating stress responses, proliferation, immune evasion, and cell migration. (B) In neurological diseases, GEF dysregulation (e.g., BIG1, Ephexin5) contributes to neuroinflammation, neurodegeneration, and impaired trafficking. (C) In cardiovascular diseases, GEFs (e.g., ARHGEF1, LARG) activate Rho GTPases (e.g., RhoA), controlling leukocyte adhesion, vascular inflammation, and smooth muscle contraction. (D) In immune diseases, GEFs (e.g., DOCK8, DOCK2) regulate immune functions, cytokine production (e.g., IL‐31, IFN‐β), and barrier dysfunction, contributing to conditions like atopic dermatitis and SLE. *Abbreviations*: PDAC (pancreatic ductal adenocarcinoma), SLE (systemic lupus erythematosus), IBD (inflammatory bowel disease), PTCL (peripheral T‐cell lymphoma), CRC (colorectal cancer), BCL (B‐cell lymphoma), HCC (hepatocellular carcinoma), NSCLC (non‐small cell lung cancer), ALL (acute lymphoblastic leukemia), MG (myasthenia gravis), HIES (hyper‐IgE syndrome), EBV‐HLH (Epstein–Barr virus‐associated hemophagocytic lymphohistiocytosis), EAE (experimental autoimmune encephalomyelitis), DIDS (specific immunodeficiency syndrome), AD (Alzheimer's disease), PD (Parkinson's disease), ALS (amyotrophic lateral sclerosis), ASD (autism spectrum disorder), HCM (hypertrophic cardiomyopathy), AAD (acute aortic dissection), PH (pulmonary hypertension), and IRI (ischemia–reperfusion injury). Figures created with BioRender.com.

GEF proteins orchestrate extraordinarily complex regulatory networks during cancer initiation and metastatic progression, involving intricate multilayered molecular interactions and extensive cross‐talk between signaling pathways (Figure 4). The Vav family exemplifies this complexity, where aberrant Vav1 expression in pancreatic ductal adenocarcinoma (PDAC) synergizes with KRAS mutations to drive malignant transformation. Mechanistically, the KRASG12D mutant stabilizes Vav1 protein through ERK‐dependent phosphorylation, extending its half‐life from 3 to 9 h. Reciprocally, activated Vav1 enhances Rac1–GTP loading efficiency by approximately eightfold via its DH domain, establishing a potent positive feedback loop [202]. This molecular interplay dramatically accelerates the progression from pancreatic intraepithelial neoplasia (PanIN) to invasive carcinoma in KPC (LSL–KrasG12D/+; Trp53flox/+; Pdx1–Cre) mouse models. Histopathological analyses reveal that Vav1‐overexpressing mice develop extensive liver metastases by 6 months (87% incidence vs. 32% in controls). Notably, Vav1's prometastatic activity exhibits striking subcellular compartmentalization: at invasion fronts, Vav1 binds Ezrin through its C‐terminal SH3 domain, localizing to pseudopodia where it activates the Rac1–PAK1 axis to drive cytoskeletal reorganization and basement membrane degradation [203].

In hematologic malignancies, Vav1 demonstrates distinct oncogenic mechanisms. Approximately 15% of peripheral T‐cell lymphoma (PTCL) patients harbor Vav1 gene rearrangements, predominantly the Vav1–THAP4 fusion variant. This chimeric protein loses Vav1's N‐terminal autoinhibitory domain while acquiring THAP4's nuclear localization signal [6, 204, 205]. Comprehensive coimmunoprecipitation–mass spectrometry analyses revealed that the nuclear‐localized fusion protein forms a transcriptional repressor complex with HDAC3 and DNMT1, specifically silencing tumor suppressor genes (e.g., CDKN1A and PTEN promoters exhibit 35–60% increased methylation) [6]. This epigenetic reprogramming confers sustained proliferative capacity. Clinical data demonstrating markedly different therapeutic responses: Vav1‐fusion patients show only 28% response to standard CHOP regimen compared with 63% objective response rate with HDAC inhibitor Romidepsin combination therapy [206] (Table 2).

**TABLE 2:** GEFs in human diseases: mutations, mechanisms, and therapeutic strategies.

GEF	Disease	Gene modulation	Mechanism	Therapeutic	References
SOS1	Noonan syndrome, KRAS‐mutant cancers	Mutations (e.g., E108K) or overexpression; BCR–ABL fusion in leukemia	Constitutive RAS activation; PH domain‐mediated interaction with BCR–ABL enhances RAS/RAC1 signaling	SOS1 inhibitors (BI‐3406, BAY‐293) PROTAC degraders (ZZ151) Combined with MEK inhibitors	[207, 208, 209, 210]
VAV1	PTCL	Gene rearrangements (e.g., VAV1–THAP4 fusion	Loss of autoinhibition; nuclear localization drives epigenetic silencing (HDAC3/DNMT1 complex)	HDAC inhibitors (Romidepsin) in VAV1‐fusion PTCL	[6, 204, 205, 206]
GEF–H1	HCC, SLE, vascular leakage	Overexpression (HCC; phosphorylation (Ser886) in endothelial dysfunction	Microtubule release → RhoA/ROCK activation (metastasis) TNF‐α/p38MAPK → barrier disruption	ROCK inhibitors (Fasudil) Microtubule stabilizers (Taxol) Peptide inhibitor (TAT‐P5)	[24, 196, 211]
DOCK8	Hyper‐IgE syndrome	Loss‐of‐function mutations	Impaired Rac/Cdc42 activation → defective immune cell migration and Treg function	HSCT	[192, 193, 194]
TRIO	Uveal melanoma	Phase separation (S1332 phosphorylation by integrin β1‐FAK)	Concentrates Rac1/WAVE components → hybrid migration	Collagen density modulation (potential target)	[212]
ARHGEF1	Hypertension, atherosclerosis	Phosphorylation (Tyr738 by Jak2	RhoA hyperactivation → vascular smooth muscle contraction	Jak2 inhibitors (preclinical)	[213]
BLOC‐3	HPS	Mutations in HPS1/HPS4	Defective Rab32/38 activation → lysosome‐related organelle dysfunction	No direct therapy; symptomatic management	[159, 160, 161, 162, 163, 164]

Abbreviations: HCC: hepatocellular carcinoma; HSCT: hematopoietic stem cell transplantation; PTCL: peripheral T‐cell Lymphoma; SLE: systemic lupus erythematosus.

GEF–H1, a crucial member of the GEFs, plays indispensable roles in cancer progression through its conserved GEF activity [214, 215, 216, 217]. Originally identified as the murine homolog Lfc from a hematopoietic cDNA library, its transforming activity requires intact GEF and PH) domains. Truncated Lfc variants lacking the microtubule‐binding C‐terminal domain promote oncogenic transformation via RhoA hyperactivation, with GEF–H1 overexpression consistently observed in H‐Ras‐transformed cells [216]. The N‐terminal C1 domain critically regulates both microtubule binding and autoinhibition—NIH‐3T3 xenografts expressing C1‐deleted GEF–H1 mutants form tumors within 9 weeks, demonstrating that removal of inhibitory sequences potently triggers transformation [43, 60, 218].

Emerging evidence indicates that noncoding RNAs (ncRNAs) can bypass C1‐mediated constraints to activate GEF–H1/RhoA signaling, coordinating stress fiber formation and focal adhesion assembly to drive metastatic dissemination [219, 220]. In HCC, elevated GEF–H1 expression strongly correlates with poor clinical outcomes [8], where it enhances cellular motility through RhoA–ROCK–myosin light chain (MLC) pathway activation [217] (Table 2). SEPT11 further amplifies this pathway via coordinated activation of RhoA/LIMK/cofilin and FAK/Src cascades to promote invasive behavior [221]. Paradoxically, TGF‐β induces GEF–H1 proteasomal degradation in NMumG cells, downregulating RhoA to initiate EMT [222, 223, 224]—demonstrating a context‐dependent duality where TGF‐β suppresses RhoA during cell detachment but activates it during migratory phases [225, 226, 227].

In colorectal cancer (CRC), the m6A reader YTHDF1 amplifies GEF–H1 translation to constitutively activate RhoA, while hypoxia‐induced exosomal circ‐133 sponges miR‐133a to relieve GEF–H1 inhibition [219, 228]. Although multiple GEFs (including βPix and Tiam1) contribute to CRC pathogenesis, GEF–H1 uniquely coordinates RhoA‐dependent cytoskeletal remodeling [147]. The tumor suppressor HUNK counteracts this oncogenic program by phosphorylating GEF–H1 at Ser645 to stabilize F‐actin networks and suppress EMT—a protective mechanism frequently lost in advanced CRC [99]. These findings underscore the dual regulatory nature of GEF–H1/RhoA signaling, which remains susceptible to microenvironmental hijacking yet is constrained by tumor‐suppressive kinases (Table 2).

GEF–H1 orchestrates metastatic behavior through diverse molecular mechanisms. The oncogene hPTTG1 transactivates the GEF–H1 promoter [229], while in triple‐negative breast cancer stem cells, GEF–H1 sustains self‐renewal capacity through PKD3 regulation [230]. During EMT progression, GEF–H1 mediates critical mechanotransduction processes by facilitating YAP nuclear translocation [127] and spatiotemporal control of invadopodia formation [231, 232]. Therapeutic strategies targeting this axis show promise, with synergistic effects observed between PKD inhibition and paclitaxel treatment [230]. Ultrasound‐induced Piezo1 activation releases microtubule‐bound GEF–H1 to enhance cellular contractility via RhoA/ROCK/MLC signaling, establishing a proapoptotic feedback loop [233]. GEF–H1 also underlies drug resistance mechanisms, with Ras‐transformed cells developing MEK inhibitor insensitivity through GEF–H1 upregulation [234, 235].

Metastatic progression involves dynamic GEF regulation in response to microenvironmental cues. In HCC hypoxic niches (<5 mmHg O_2_), HIF‐1α induces 4.7‐fold upregulation of DOCK1 expression to drive metastasis through dual mechanisms: Rac1–GTP elevation (3.2‐fold increase) and PKM2‐dependent diversion of glycolytic flux to the pentose phosphate pathway [236]. Racial disparities in prostate cancer bone metastasis reveal functional consequences of the ROBO1 rs10892563 SNP (MAF = 0.37 in African Americans), which disrupts CTCF binding to derepress DOCK1 expression, enhancing SLIT2‐resistant osteolytic activity [237].

Spatiotemporal regulation is exemplified by Trio's phase separation behavior in uveal melanoma. Elevated collagen density (>5 mg/mL) triggers integrin β1‐FAK‐mediated Trio phosphorylation at S1332, promoting liquid–liquid phase separation of the SR7 domain to concentrate Rac1/WAVE components (15× enrichment) [212]. BAP1 loss mislocalizes nuclear Trio, creating epigenetic imbalance in Rho GTPase regulation through ARHGAP18 upregulation (3.5×) and ARHGEF4 silencing [238], thereby enabling hybrid migration modes.

Recent structural insights have advanced understanding of SOS family regulation. Cryo‐EM analyses demonstrate that KRASG12C inhibitors disrupt critical SOS1 salt bridges (increasing Y884–R73 distance from 2.8 to 4.5 Å), reducing GEF activity by 1000‐fold [239]. The PROTAC ZZ151 (featuring a 12‐carbon linker) achieves 3.2 nM binding affinity (*K*_d_), sustaining SOS1 degradation in KRASG12D GEMMs with 85% pERK suppression [240]. SOS2 compensation occurs via mTORC1–S6K1‐mediated Ets activation, preferentially signaling through HRAS–PI3K—a vulnerability effectively targeted by SOS1/PI3Kβ combination therapy (7.3‐month progression‐free survival; 64% objective response rate in SOS1/SOS2 high tumors) [241].

Emerging noncanonical GEF functions include Vav2's regulation of the mevalonate pathway: nuclear Vav2 binds SREBF1 (*K*_d_ = 8.3 nM) to transactivate HMGCS1 (4.8× induction), while cytoplasmic Vav2 stabilizes HMGCR (extending half‐life from 2 to 6 h). SOS1 loss induces extensive H3K27me3 reprogramming in KRASG12D PDAC, erasing ductal identity markers (Krt19↓3.5×) while restoring acinar differentiation markers [242, 243].

Phase separation fundamentally underpins metastatic signaling hubs. Trio SR7 droplets (forming at pH < 6.5) concentrate promigratory factors (WAVE2↑15×) while excluding RhoGDI, with dramatically reduced diffusivity (D↓100×) enabling sustained signal persistence. Clinically, droplet formation frequency strongly predicts metastatic potential (hazard ratio = 3.2) [212].

In summary, GEF–H1 and related family members critically govern tumor migration, proliferation, mitotic progression, and therapeutic resistance, making their precise regulation a compelling therapeutic target. These mechanistic insights unveil multifaceted targeting opportunities—ranging from enzymatic inhibition to phase separation disruption—while simultaneously highlighting the challenge of integrating these complex mechanisms into robust precision oncology frameworks. Future research must address the contextual dependencies of GEF regulation across different tumor types and microenvironments to realize the full therapeutic potential of targeting these master regulators of cancer progression.

### 5.2 GTPase Dysregulation in Neurodegenerative Diseases

Extensive research has established that Rab3 and Syt family proteins are critical regulators of synaptic vesicle fusion and neurotransmitter release. Rab3, a small GTPase, orchestrates vesicle trafficking, docking, and fusion through its GTP/GDP cycling (the “Rab cycle”), while Syt acts as a high‐affinity calcium sensor, directly mediating calcium‐dependent synchronous neurotransmitter release. These systems function synergistically to ensure neuronal signaling fidelity, and their dysregulation is implicated in multiple neurodegenerative disorders [244].

In neuroinflammatory contexts, biochemical studies have identified BFA‐inhibited guanine nucleotide‐exchange protein 1 (BIG1) as a key modulator of microglial activation, regulating both inflammatory cytokine production and migratory capacity via the PI3K/Akt/NF‐κB cascade. Notably, BIG1 expression strongly correlates with proinflammatory mediator secretion (e.g., TNF‐α, IL‐1β, IL‐6), highlighting its potential as a therapeutic target for neuroinflammation‐associated degeneration [245].

At the molecular level, neurodegeneration is tightly regulated by Rho GTPase signaling networks, which govern neuronal survival, synaptic plasticity, and cytoskeletal dynamics. E5, a neuronal RhoGEF with selectivity for RhoA and Cdc42, exhibits activity‐dependent modulation via synaptic inputs and tyrosine phosphorylation [246]. In Angelman syndrome models, E5‐mediated RhoA hyperactivation drives synaptic elimination and cognitive deficits. Conversely, activity‐dependent synaptic strengthening involves a switch to Cdc42 activation, promoting synapse stabilization [246]. This phosphorylation‐regulated duality positions E5 as a molecular switch in neurodegeneration. In amyotrophic lateral sclerosis, Rho GTPase homeostasis is severely disrupted: SOD1–G93A transgenic mice exhibit reduced Rac1‐GTP levels and aberrant nucleo‐axonal RhoB redistribution—changes hypothesized to drive motor axon retraction  [247]. Compounding this effect, microglial Rac1 overactivation generates neurotoxic NADPH oxidase‐derived ROS, exacerbating motor neuron injury.

A recent breakthrough identified CalDAG–GEFI (CDGI) as the physiological GEF modulating LRRK2 GTPase activity in neurodegeneration. Although LRRK2 mutations (a major cause of familial Parkinson's disease (PD)) affect a multidomain protein with both GTPase and kinase functions, prior research disproportionately focused on kinase activity, leaving GTPase regulation poorly understood. Structural analyses reveal that CDGI binds LRRK2's ROC domain via a canonical GEF module, catalyzing GDP/GTP exchange in a manner dependent on LRRK2's nucleotide‐binding pocket integrity. Functionally, CDGI enhances LRRK2's GTP‐binding capacity, membrane association, and kinase activity (evidenced by increased Rab10 phosphorylation—effects amplified by calcium signaling, implicating calcium dyshomeostasis in LRRK2‐linked PD pathogenesis. Genetic evidence from Drosophila and murine models confirms CDGI's role in LRRK2‐dependent neurodegeneration: (1) progressive retinal degeneration in flies, and (2) striatal dopaminergic neuron loss with motor deficits in mice—phenotypes abolished in LRRK2‐null backgrounds, demonstrating strict LRRK2 dependence [248].

Neurodegenerative diseases are characterized by progressive neuronal dysfunction and death, stemming from dysregulated cellular and molecular homeostasis. AD, the most prevalent neurodegenerative disorder, is pathologically defined by extracellular β‐amyloid plaques and intraneuronal hyperphosphorylated Tau accumulation. Emerging evidence implicates lysosomal dysfunction as another key pathogenic factor. Lysosomal positioning and function, regulated by small GTPases (e.g., Rab, Arl8b), require precise molecular control. Recent studies identified DENND6A, a Rab‐specific GEF that activates Rab34 to recruit the RILP–dynein complex, facilitating retrograde lysosomal transport [249]. Notably, DENND6A deficiency disrupts lysosomal distribution and impairs autophagic flux, potentially accelerating protein homeostasis collapse and neurodegeneration [249].

### 5.3 RhoGEFs in Vascular Inflammation and Remodeling

Cardiovascular pathology encompasses the molecular mechanisms and structural alterations underlying various heart and vascular diseases. The GEF proteins play a central role in inflammation, vascular function regulation, and myocardial remodeling through modulation of the Rho/Rac signaling pathway.

The VAV family exhibits pleiotropic functions in cardiovascular pathologies. Trans‐anethole mitigates myocardial ischemia–reperfusion injury and enhances cardiac function by upregulating VAV3 mRNA and its epigenetic regulators miR‐1298 and lncRNA JRKL–AS1, thereby suppressing the proinflammatory cytokine TNF‐α [250]. VAV3 and VAV3–AS1 polymorphisms correlate with hypertension risk by promoting VSMC proliferation and migration via Rho/Rac signaling [251]. In atherosclerosis, VAV proteins regulate CD36 receptor endocytosis, thereby modulating oxidized LDL (oxLDL) uptake and JNK kinase activation. Notably, Vav2 deficiency reduces oxLDL uptake and attenuates atherosclerosis progression [252]. Moreover, Vav2 enhances nitric oxide (NO)‐mediated vasodilation by inhibiting phosphodiesterase 5 via the Rac1/Pak1 pathway. Vav2‐deficient mice exhibit impaired vasodilation, leading to hypertension and cardiovascular remodeling [253].

The ARHGEFs, including LARG and ARHGEF1, are critical in vasoconstriction and hypertension. Angiotensin II (Ang II) upregulates LARG via AT1 receptor activation, stimulating the RhoA/Rho kinase/MYPT1 axis to promote VSMC contraction and increased vascular tone [254]. In spontaneously hypertensive rats (SHRs), baseline LARG expression in VSMCs is elevated, and while Ang II further upregulates LARG in normotensive rats, no additional increase occurs in SHRs, suggesting a role in calcium‐sensitive vasoconstriction via RhoA/ROCK signaling [255] (Table 2).

ARHGEF1 also contributes to vascular inflammation and atherosclerosis. Ang II enhances leukocyte–endothelial adhesion via ARHGEF1, and ARHGEF1‐deficient mice show reduced leukocyte recruitment and atherosclerosis, implicating ARHGEF1 in β2 integrin regulation [256]. Furthermore, Jak2‐mediated phosphorylation of ARHGEF1 at Tyr738 activates RhoA, increasing VSMC contractility and vascular resistance—a key mechanism in hypertension [213].

In myocardial pathology, PDZ–RhoGEF (ARHGEF11) is upregulated in hypertrophic hearts and promotes pathological cardiac hypertrophy via RhoA‐dependent signaling and Gα13 interaction [257]. Meanwhile, Ect2, a cytokinesis regulator, enhances cardiomyocyte proliferation but impairs contractility when overexpressed with PIK1 (T210D)  [258]. p63RhoGEF contributes to VSMC contraction by activating RhoA/ROCK, inhibiting myosin light chain phosphatase (MLCP)  [259].

TIAM1, a Rac1‐specific GEF, strengthens endothelial barrier integrity via optogenetic activation (Opto‐RhoGEFs), even after VE‐cadherin blockade  [260]. In aortic dissection (AAD), S‐nitrosylated Septin2 in macrophages relieves TIAM1 inhibition, activating RAC1–NF‐κB to promote vascular inflammation. RAC1 inhibitors (NSC23766, R‐Ketorolac) attenuate AAD progression [198]. Additionally, a BASP1‐high monocyte subset exacerbates AAD via PIP2–SP1–ACTN1/VAV3 and ITGB1–Rac1–GSN pathways, differentiating into proinflammatory BASP1+ macrophages  [261].

ROS contribute to cardiovascular pathology via NADPH oxidase and mitochondrial dysfunction, activating ARHGEF1 and Src kinases to induce RhoA‐mediated vasoconstriction—a key mechanism in hypoxic pulmonary hypertension [262]. Endothelial dysfunction, marked by reduced NO bioavailability, arises from eNOS uncoupling due to BH_4_ oxidation, l‐arginine deficiency, and ADMA accumulation. Strategies restoring eNOS coupling (e.g., BH_4_/folate supplementation) may improve endothelial function [263].

GEF proteins critically regulate inflammation, vascular tone, myocardial remodeling, and endothelial function via Rho/Rac signaling. These insights not only elucidate cardiovascular disease mechanisms but also highlight potential GEF‐targeted therapies (e.g., against VAV3, ARHGEF1, TIAM1, or PDZ‐RhoGEF) for hypertension, atherosclerosis, and myocardial regeneration. The mechanistic overlap between cardiovascular diseases and cancer (e.g., shared roles of ARHGEF12, CTNNB1, and PTEN) may inform dual therapeutic strategies [264].

### 5.4 Immunological Disorders: Vav/Dock Family Dysfunction

The GEF proteins play multifaceted regulatory roles in immune pathogenesis through their modulation of Rho GTPases, with individual members exhibiting both specialized and overlapping functions. Notably, among these, the Vav family (Vav1–3) serves as canonical GEFs with cell‐type‐specific actions: In macrophages, Vav1 deficiency selectively impairs migration speed without altering the enhanced membrane ruffling phenotype observed in Vav2‐deficient cells, though both reduce adhesion area through mechanistically distinct pathways [265]. Within T cells, Vav1 demonstrates dual functionality as both a GEF and adaptor protein, critically regulating TCR signaling and thymic development. Genetic ablation of Vav1 markedly attenuates autoreactive T cell responses in experimental autoimmune encephalomyelitis models [266] and synergizes with Themis to potentiate T cell activation via inhibition of the phosphatase SHP‐1 [189]. Clinically relevant polymorphisms include the Vav1 R63W variant, which predisposes to myasthenia gravis through enhanced IFN‐γ/IL‐17A production [267], while Vav3 exhibits noncanonical nuclear functions in B‐cell acute lymphoblastic leukemia (B‐ALL) by sustaining epigenetic silencing through PRC1 complex interactions [268].

The DOCK family presents a paradigm of functional divergence despite structural conservation: While DOCK2 and DOCK8 both possess DHR‐2 domains, their deficiencies yield distinct immunological phenotypes. DOCK2 impairment predominantly disrupts Rac activation, manifesting as CID with neutrophil dysfunction [269]—a phenotype amenable to pharmacological inhibition via CPYPP‐mediated blockade of T cell migration [270]. In contrast, DOCK8 mutations dysregulate Cdc42 signaling, resulting in hyper‐IgE syndrome characterized by defective Tfh cell homing [271], metabolic exhaustion of IgA⁺ plasma cells [272], and pathogenic Treg accumulation [273] (Table 2). Recent studies reveal DOCK2's nonredundant role in antiviral immunity, where its deficiency specifically compromises CD8⁺ T cell clonal expansion during HSV‐1 infection [274].

GEF–H1 exemplifies functional pleiotropy in immune dysregulation: In systemic lupus erythematosus (SLE), PPP2R2A‐mediated dephosphorylation of GEF–H1 activates the RhoA–ROCK axis, driving pathogenic IL‐17/IFN‐γ production in T cells [26] (Table 2). Its inflammatory roles extend to inflammatory bowel disease (IBD), where it compromises intestinal epithelial barrier integrity [275], while in antiviral defense, it potentiates IFN‐β generation through TBK1–IKKε pathway activation [276]. This functional spectrum complements Vav1's centrality in adaptive immunity. Together, they illustrate how GEF–H1 and Vav1 contribute to SLE pathogenesis through innate and adaptive mechanisms, respectively. Additional GEF members demonstrate cooperative regulation, exemplified by P‐Rex's collaboration with Vav in neutrophil pulmonary trafficking [277], while RhoH deficiency impacts T cell development via dysregulated ZAP‐70/Lck localization and associates with both SLE and B‐cell malignancies [278].

Therapeutic targeting strategies reflect this functional diversity: Molecular interventions include DOCK2‐specific inhibitors and emerging Vav1‐directed compounds for B‐ALL [279]. Immunomodulatory approaches encompass IL‐6 blockade to mitigate DOCK8 deficiency‐associated inflammation [280] and CD147–Vav1–Rac1 axis modulation to control macrophage hyperactivation [281]. HSCT remains curative for DOCK family deficiencies, though requiring careful monitoring for complications such as EBV‐associated hemophagocytic lymphohistiocytosis [282].

This integrated analysis reveals a hierarchical regulatory network: The Vav family orchestrates adaptive immune signaling, DOCK proteins coordinate cellular motility and metabolic homeostasis, while GEF–H1 bridges innate and adaptive immunity. Such multilayered governance provides a conceptual framework for both mechanistic classification and precision therapeutics in immune disorders.

## 6 Therapeutic Targeting Strategies

After an in‐depth investigation into the regulatory mechanisms of GEFs in diseases, directly inhibiting their catalytic activity has emerged as a central focus of therapeutic strategies. By targeting the catalytic domain of GEFs, researchers have developed a series of small‐molecule inhibitors designed to block the interaction between GEFs and GTPases, thereby precisely modulating downstream signaling pathways. These inhibitors not only provide novel therapeutic avenues for diseases such as cancer but also lay the groundwork for further exploration of alternative strategies, such as disrupting protein–protein interaction interfaces (Table 3).

**TABLE 3:** Therapeutic targeting strategies for GEFs.

Strategy	Drugs/methods	Mechanism of action	Indications	References
Direct Inhibition of GEF catalytic activity	BI‐3406, BAY‐293 (SOS1 inhibitors)	Block SOS1–KRAS interaction, inhibiting RAS signaling	KRAS‐mutant cancers Metastatic cancers	[207, 208, 209, 210]
	NSC23766 (Tiam1 inhibitor	Inhibits Tiam1–Rac1 interaction, suppressing metastasis	Diabetes, prostate dysfunction	[283]
	SecinH3 (Arf–GEF inhibitor	Targets the Sec7 domain, inhibiting Arf6 activation	Breast cancer, leukemia	[284]
	ZINC69391 (P‐Rex1 inhibitor	Binds the DH domain, blocking Rac1 activation		[285]
Disruption of protein–protein interactions	Phosphopeptide bZAP 1.1 (Vav1 inhibitor	Binds Vav1's SH2 domain, disrupting ZAP‐70 signaling	Immune dysregulation	[286]
	Modified peptides (Tiam1–Rac1 interface	Mimics Tiam1–Rac1 binding, inhibiting migration	Cancer metastasis	[287]
	Y16 (LARG inhibitor	Targets the DH–PH junction, suppressing RhoA activation	Hypertension, cardiovascular diseases	[288]
PROTAC technology	ZZ151, BTX‐6654 (SOS1 degraders	Induces SOS1 ubiquitination and degradation, overcoming KRAS inhibitor resistance	KRAS‐mutant resistant tumors	[210, 289]
	MRT‐6160 (Vav1 degrader	Molecular glue degrades Vav1, suppressing T‐cell hyperactivation	Autoimmune diseases	[188]
CRISPR screening	SOS1/SOS2 dual knockout	Identifies compensatory mechanisms, guiding dual‐target PROTAC design	KRAS‐mutant cancers	[290]
	DOCK8 gene editing	Restores immune cell migration in DOCK8 deficiency	DOCK8 deficiency syndrome (HIES)	[192, 193, 194]
Combination therapies	SOS1 inhibitor + MEK inhibitor	Prevents RAS–MAPK feedback reactivation	KRAS‐mutant cancers	241, 291
	ROCK inhibitor (Fasudil) + microtubule stabilizer (Taxol)	Synergistically blocks GEF–H1 release and RhoA–ROCK signaling	Metastasis, vascular dysfunction	[211]

### 6.1 Direct Inhibition of GEF Catalytic Activity

In recent years, breakthrough progress has been made in therapeutic strategies targeting the catalytic activity of GEFs for disease treatment. As crucial regulators of small GTPase activity, GEFs play pivotal roles in diverse cellular signaling pathways, and their aberrant activation is closely associated with various pathological processes including tumorigenesis, inflammatory responses, and metabolic disorders. Based on the classification characteristics of GEFs, researchers have developed multiple specific inhibition strategies, providing novel approaches for targeted therapy of related diseases.

In the regulation of RAS signaling pathways, SOS family GEFs have attracted considerable attention due to their central role in RAS activation. Studies have demonstrated that SOS1 and SOS2 play critical roles in various malignancies by catalyzing GDP/GTP exchange of RAS proteins. Targeting this mechanism, researchers have developed several small‐molecule inhibitors, including BI‐3406, BAY‐293, and BI‐170192, which effectively block SOS1–KRAS interaction through specific binding to the CDC25 catalytic domain of SOS1 [207, 208] (Table 3). Besides directly targeting catalytic activity, allosteric modulation strategies have shown promising potential. Interventions at the REM domain of SOS1 or designed mutations at specific allosteric sites (e.g., L687E/R688A) can significantly disrupt its positive feedback loop with RAS–GTP [209]. With advances in protein degradation technology, PROTAC molecules such as ZZ151 and 89 have demonstrated enhanced anti‐proliferative activity in KRAS‐mutant tumor models by inducing SOS1 ubiquitination and degradation [210]. For clinical translation, combination therapies have shown unique advantages, with SOS1 inhibitors exhibiting significant synergistic effects when combined with MEK inhibitor trametinib or KRAS G12C inhibitor adagrasib [292, 293]. Additionally, inhibitors targeting other RAS–GEFs like RasGRP1, such as catechin hydrate (CH), have been confirmed to regulate macrophage inflammatory responses through direct binding [294].

In the regulation of Rho family GEFs, researchers have identified multiple therapeutically valuable inhibition mechanisms. The autoinhibitory property of AKAP–Lbc provides novel insights for drug design, as its N‐ and C‐terminal domains maintain an inactive state through intramolecular interactions, particularly when Ser1565 phosphorylation enhances 14‐3‐3 protein binding [295, 296]. Recently, various small‐molecule inhibitors targeting different Rho GEF members have been successfully developed: NSC23766 effectively inhibits tumor metastasis by specifically blocking Tiam1–Rac1 interaction [283]; ZINC69391 and its derivative 1A‐116 exert inhibitory effects by targeting the DH domain of P‐Rex1 [285]; while intervention at the PH domain of Ect2 has been proven to effectively disrupt its binding with RhoA [297] (Table 3). Beyond direct targeting of catalytic activity, interfering with GEF subcellular localization represents another effective strategy. For instance, inhibiting PIP3 interaction with the PH domain of P‐Rex1 prevents its membrane localization and activation [298]. In disease models, peptide inhibitor TAT–P5 demonstrates remarkable efficacy in alleviating fibrosis and improving endothelial barrier function through specific targeting of GEF–H1 [211].

Inhibition strategies for Arf family GEFs exhibit unique advantages in treating metabolic diseases. SecinH3, a specific inhibitor of cytohesin‐2 (ARNO), effectively suppresses Arf6 activation by targeting its Sec7 domain, showing potential applications in diabetes and prostate smooth muscle dysfunction [284]. The classical inhibitor BFA blocks nucleotide exchange by stabilizing a unique conformation of the Arf–GDP/ArfGEF complex [299]. Notably, although initially designed as an Arf6 inhibitor, NAV‐2729 was later found to exhibit stronger inhibitory activity against BRAG2 [299]. In bone metabolism regulation, the DOCK family member DOCK5 negatively regulates osteogenic differentiation through Rac1 activation, and its specific inhibitor C21 significantly promotes bone tissue regeneration [300]. Additionally, strategies disrupting DOCK–ELMO complex formation have been confirmed to effectively inhibit excessive Rac signaling activation [301].

The critical roles of Vav family GEFs in various diseases have also drawn extensive research attention. In HIV‐associated nephropathy, VAV2 promotes podocyte injury through Rac1 signaling activation, with knockout experiments confirming that VAV2 inhibition significantly improves pathological alterations [302]. In hematologic malignancies, the development of VAV3‐specific inhibitor IODVA1 provides new solutions for overcoming tyrosine kinase inhibitor resistance [303]. Despite significant progress in GEF inhibitor development, numerous challenges remain, particularly the selectivity issues arising from high structural similarity among family members, as exemplified in SOS2 inhibitor development [304]. To address these challenges, combination therapies demonstrate unique advantages. For instance, combining SOS1 inhibitors with MEK inhibitors effectively blocks feedback activation of signaling pathways [291]. Emerging technologies like PROTAC and allosteric modulation provide novel approaches for targeting traditionally “undruggable” targets [241].

In conclusion, classification‐based specific inhibition strategies targeting GEF families have opened new avenues for targeted therapy of various diseases. From inhibiting catalytic activity to modulating protein interactions, and from intervening in subcellular localization to inducing protein degradation, diverse intervention approaches provide abundant options for precision medicine.

### 6.2 Disruption of PPI Interfaces

PPIs serve as fundamental mechanisms underlying cellular life activities, participating in the regulation of key biological processes including signal transduction, gene expression regulation, and cell cycle progression. In recent years, with advancements in structural biology and computational biology, targeting PPI interfaces has emerged as a novel therapeutic strategy for various diseases. Particularly in studies of GEF proteins, therapeutic approaches disrupting specific PPI interfaces have demonstrated significant clinical potential. GEF proteins regulate crucial physiological and pathological processes such as cell proliferation, migration, and differentiation through activation of small GTPases (e.g., Ras and Rho families), with their aberrant activation being closely associated with tumorigenesis and immune disorders. Research has revealed that although DOCK2 lacks the canonical c‐Crk‐II binding motif, it can specifically interact with the SH3 domain of CrkL through two independent regions [305]. This discovery not only demonstrates the existence of nonclassical binding modes but also provides a theoretical foundation for developing small‐molecule inhibitors targeting SH3 domains. Notably, disruption of the dCS region of DOCK2 or use of dominant‐negative mutant DOCK2–dCS significantly inhibits Rac1 activation [305], showing potential therapeutic value for leukemia and immune diseases.

In studies of Vav family proteins, scientists have identified that the interaction between their SH2 domain and phosphorylated tyrosine residues of ZAP‐70, constitutes a critical step in signal transduction. Interestingly, synthetic phosphorylated peptide bZAP 1.1 can effectively block this interaction through competitive binding [286]. Further research demonstrated that simultaneous targeting of both Vav1 and Vav3 yields more pronounced therapeutic effects than single inhibition due to their functional redundancy in platelet activation [306]. Mechanistically, the acidic region of Vav1 (containing key residue Tyr174) maintains an autoinhibited state through intramolecular interaction with the DH domain, while phosphorylation of this residue releases inhibition and promotes Rac binding [307]. Additionally, introducing SH2 domain mutations (R694K) or using competitive fragment Vav‐C can effectively inhibit Vav1 membrane localization [308], providing important insights for developing novel immunomodulators.

Regarding regulatory mechanisms, SOCS1 promotes Vav ubiquitination and degradation through SH2 domain‐mediated binding to the Vav acidic region [309]. Similarly noteworthy, the interaction between Vav1 and SLP‐76 depends on the SH2 domain, with specific mutations (e.g., R697L) significantly suppressing NFAT activation [310]. These findings not only deepen our understanding of immune signaling pathways but also identify new therapeutic targets.

In Tiam1 studies, researchers discovered that its PH domain recognizes specific lipid molecules through a noncanonical phosphoinositide‐binding pocket. Site‐directed mutagenesis of key residues (e.g., R459A/R460A) markedly impairs membrane localization [311]. Particularly, the DH–PH domain of Tiam1 exhibits higher catalytic efficiency toward membrane‐bound Rac–GDP, suggesting that disrupting Rac–GDI interaction may enhance GEF activity [312]. Based on structural insights, modified peptides mimicking the Tiam1–Rac1 interaction interface were designed and demonstrated effective inhibition of Rac1–GTP levels [287].

Significant progress has been made in understanding P‐Rex1 regulation. Its catalytic activity is finely modulated by DH‐DEP1 and PH‐4HB domain interactions, with specific mutations (e.g., L177E) substantially increasing GEF activity [313]. In drug development, small‐molecule compound PREX‐in1 effectively blocks P‐Rex1–Rac binding by specifically targeting the DH domain [298]. Notably, PI3K inhibitors can suppress ERK pathway activation by inhibiting P‐Rex1–Rac1 interaction [314], providing experimental support for combination therapies (Table 3).

In KRAS signaling research, the Sos1–KRAS interaction represents an important therapeutic target. Small molecules like BAY‐293 and BI‐3406 effectively disrupt this interaction [315]. Emerging PROTAC technology (e.g., ZZ151) demonstrates superior efficacy by inducing Sos1 degradation [210]. Structural studies revealed that Noonan syndrome‐associated mutations (e.g., E108K) relieve Sos1 autoinhibition [316], offering new perspectives for understanding disease mechanisms and drug development.

Among RhoGEF proteins, p115–RhoGEF exhibits unique regulation. Its RH‐DH linker region suppresses GEF activity, which can be relieved by Gα13 binding or specific mutations [317]. Compound Y16 effectively blocks RhoA activation by targeting the DH–PH junction of LARG [288]. Structural analysis identified key residues (e.g., E423, R551) at the ARHGEF1–RHOA interface as promising targets for inhibitor development [318].

PDZRhoGEF studies revealed autoinhibition through interaction between its negatively charged sequence (e.g., Asp706) and the DH domain, with charge‐reversal mutations (e.g., D706R) significantly enhancing catalytic activity [319]. PLD2 research demonstrated that its PH domain can serve as a noncanonical GEF for Rac2, with specific mutations (e.g., Δ263–266) effectively inhibiting Rac2 activation [320], expanding our understanding of GEF functional diversity.

Despite the inherent challenges in targeting PPI interfaces, which are typically flat and dynamic, recent advances in computational approaches and fragment‐based drug discovery [321] have provided powerful tools for rational drug optimization. These methodological breakthroughs not only deepen our mechanistic understanding of cellular signaling pathways but also create a robust theoretical framework for developing innovative therapeutics against cancer and immune‐related diseases.

### 6.3 Emerging Technologies: PROTACs and CRISPR Screens

PROTAC technology and CRISPR screening, as cutting‐edge biomedical tools, exhibit powerful synergistic effects in studies related to the GEFs. PROTAC technology achieves remarkable results in targeting GEFs such as SOS1 through the design of bifunctional molecules to induce targeted protein degradation. Multiple studies have developed CRBN‐ or VHL‐based SOS1 PROTAC degraders, such as compound 11o (DC50: 1.85–7.53 nM), LHF418 (DC50: 209.4 nM), and BTX‐6654/BTX‐7312. These degraders effectively suppress the KRAS signaling pathway and overcome resistance to traditional inhibitors by inducing efficient SOS1 degradation [289, 322, 323]. CRISPR screening provides critical support for PROTAC research by systematically validating the biological significance of SOS1 through gene editing. For instance, SOS1‐knockout cells exhibit reduced sensitivity to PROTACs, while CRBN knockout completely abolishes degradation capacity, confirming the mechanism‐dependent action of PROTACs [289]. Additionally, CRISPR screening has uncovered functional redundancy and compensatory mechanisms among GEFs, such as the finding that P‐Rex1's central role in PIP3‐driven Rac activation can be compensated by other GEFs like the Vav family [324]. These insights provide a foundation for designing more effective targeting strategies.

Meanwhile, in immune system‐related GEF research, CRISPR screening plays a pivotal role. Studies reveal that VAV1, a key regulator of T‐cell receptor signaling, significantly suppresses T‐cell activation and cytokine secretion upon knockout [188]. Based on these findings, researchers have begun developing molecular glue degraders targeting VAV1, such as MRT‐6160, highlighting PROTAC technology's potential in immunotherapy. Similarly, CRISPR screening combined with whole‐exome sequencing identified an ARHGEF12 SNP (rs10892563) influencing erythrocyte regeneration via the RhoA–p38 pathway [325], offering novel therapeutic insights for anemia. These studies demonstrate that CRISPR screening efficiently identifies GEFs functions and their disease relevance, while PROTAC technology provides a direct tool for functional intervention.

Other GEFs, such as RABIF/MSS4 and SOS2, have gained new functional understanding through CRISPR technology. Genome‐wide CRISPR screening unexpectedly revealed that RABIF/MSS4 does not act as a conventional GEF but instead stabilizes Rab GTPases by preventing Rab10 proteasomal degradation, thereby regulating GLUT4 exocytosis [326]. This discovery expands the understanding of Rab GTPase regulation and suggests new directions for PROTAC development targeting membrane trafficking. SOS2 knockout studies demonstrate its specific regulation of the PI3K/AKT pathway in KRAS‐mutant tumors, functionally complementing SOS1 [290], implying that dual‐target PROTACs degrading both SOS1 and SOS2 may yield synergistic therapeutic effects (Table 3).

The integration of PROTAC and CRISPR technologies creates a closed‐loop research framework for GEFs studies: CRISPR screening systematically identifies disease‐associated GEF members and their regulatory networks (e.g., SHP2 [encoded by *PTPN11*] as a critical mediator of the MAPK pathway [327], while PROTAC technology enables the design of targeted degraders for functional validation and therapeutic development. This synergy accelerates both target discovery/validation and the development of novel strategies to overcome drug resistance. For example, CRISPR screening can identify resistance mechanisms to KRAS inhibitors, guiding the design of corresponding PROTACs for precision intervention [328].

Looking ahead, advancements in PROTAC design (e.g., enhanced selectivity, improved pharmacokinetics) and CRISPR screening (e.g., reduced off‐target effects, higher throughput) will deepen the integration of these technologies, further advancing GEFs applications in disease treatment. This convergence holds promise for breakthroughs in cancer, immune disorders, and metabolic diseases [329, 330].

## 7 Challenges and Future Directions

The study of GEFs and their small GTPase targets has revolutionized our understanding of cellular signaling networks and their dysregulation in human diseases. While tremendous progress has been made in elucidating the structural basis of GEF–GTPase interactions and their physiological functions, several fundamental and translational challenges must be addressed to fully realize their therapeutic potential.

At the molecular level, the remarkable diversity of GEF isoforms presents both opportunities and challenges. For GEF–H1 in particular, the existence of multiple splice variants with potentially distinct functions remains largely unexplored territory. The development of isoform‐specific detection tools and functional assays will be crucial to dissect their individual contributions to cellular processes. Furthermore, the complex posttranslational regulation of GEFs, particularly the intricate crosstalk between phosphorylation, ubiquitination, and other modifications, represents a critical knowledge gap. Advanced proteomic approaches combined with genetically engineered models will be essential to map these regulatory networks and understand how they are rewired in disease states.

The contextual regulation of GEF activity adds another layer of complexity. Emerging evidence suggests that GEFs like GEF–H1 can integrate diverse inputs‐from mechanical forces to cytokine signals‐but the molecular mechanisms underlying this signal integration remain poorly understood. Super‐resolution microscopy and biosensor technologies may help visualize how GEFs process these signals in real‐time within living cells. Similarly, the relative contributions of microtubule‐dependent versus independent activation pathways need clarification, particularly in pathological conditions where microtubule dynamics are altered, such as in metastatic cancers or neurodegenerative disorders.

From a therapeutic perspective, the high structural conservation among GEFs poses significant challenges for drug development. Current inhibitors often lack sufficient specificity, leading to off‐target effects. Innovative approaches such as covalent inhibitors, PROTACs, or molecular glues may provide solutions to this selectivity problem. The development of such agents would be greatly facilitated by high‐resolution structural data from cryo‐EM and AI‐assisted protein modeling. Additionally, the identification of allosteric regulatory sites could offer new opportunities for targeted modulation of GEF activity.

Technological advances are opening exciting new avenues for GEF research. Single‐cell multiomics approaches will be invaluable for mapping GEF expression patterns and activity states across different cell types in health and disease. CRISPR‐based screening platforms can systematically identify genetic interactors and synthetic lethal partners of specific GEFs, revealing new therapeutic vulnerabilities. Advanced imaging modalities, including expansion microscopy and lattice light‐sheet microscopy, could provide unprecedented views of GEF localization and dynamics in intact tissues.

The tumor microenvironment presents complex challenges for GEF‐targeted therapies. The mechanical and biochemical heterogeneity of tumors likely creates distinct niches where GEF activity is differentially regulated. Understanding how factors like hypoxia, extracellular matrix stiffness, and immune cell interactions modulate GEF signaling will be critical for developing context‐specific interventions. Similarly, in neurological disorders, the cell‐type specific effects of GEF modulation in neurons versus glia need careful evaluation.

Clinical translation will require innovative combination strategies. Given the compensatory mechanisms and signaling redundancy in many pathways regulated by GEFs, rational combinations with microtubule‐targeting agents, kinase inhibitors, or immunotherapies may be necessary for durable responses. The development of predictive biomarkers will be equally important to identify patients most likely to benefit from GEF‐targeted approaches. Early‐phase clinical trials should incorporate sophisticated pharmacodynamic assessments to verify target engagement and pathway modulation.

Beyond their canonical roles as GTPase activators, GEFs may have underappreciated functions as scaffolds, adaptors, or even transcriptional regulators. Systematic investigation of these noncatalytic roles could uncover entirely new biological functions and therapeutic opportunities. Similarly, the potential crosstalk between GEFs and other signaling systems, such as metabolic pathways or the DNA damage response, remains largely unexplored.

In conclusion, while the path forward presents significant challenges, the potential rewards are substantial. By leveraging cutting‐edge technologies and interdisciplinary approaches, we can overcome current limitations in our understanding of GEF biology and translate these insights into transformative therapies. Future research should prioritize the development of more specific pharmacological tools, comprehensive characterization of GEF regulatory networks, and innovative clinical trial designs. As we continue to unravel the complexities of GEF signaling, we move closer to realizing their full potential as therapeutic targets for cancer, immunological disorders, neurodegenerative diseases, and other conditions where GEF dysregulation plays a central role. The coming decade promises to be an exciting period of discovery and translation in this dynamic field.

## Author Contributions

Z.L., C.N., H.J., J.K., and Y.Z. made contributions to the conception or design of the study. Z.L., P.L., X.L., C.N., R.W., W.L., and B.L. made the figures and tables. P.L., H.Y., J.L., Y.Y., L.D., and H.J. revised pictures and tables with input from all authors. Z.L. wrote the initial draft of the manuscript. X.W., J.R., and Y.Z. read and revised early drafts of the manuscript. All authors read and approved the final manuscript.

## Conflicts of Interest

The authors declare no conflicts of interest.

## Ethics Statement

The author has nothing to report.

## Data Availability

The data that support the findings of this study are available from the corresponding author upon reasonable request.
